# Integrated phenotypic, transcriptomics and metabolomics: growth status and metabolite accumulation pattern of medicinal materials at different harvest periods of *Astragalus Membranaceus Mongholicus*

**DOI:** 10.1186/s12870-024-05030-7

**Published:** 2024-05-03

**Authors:** Xiaojie Li, Yingtong Mu, Mei Hua, Junjie Wang, Xiaoming Zhang

**Affiliations:** 1 Engineering Research Center for the Seed Breeding of Chinese and Mongolian Medicinal Materials in Inner Mongolia, Hohhot, 010010 Inner Mongolia China; 2 Key Laboratory of Grassland Resources, College of Grassland, Resource and Environmental Science, Inner Mongolia Agricultural University, Ministry of Education, Hohhot, 010021 P.R. of China

**Keywords:** *Astragalus Membranaceus Mongholicus*, Phenotype, Isofavone and triterpenoid saponins substances accumulation, Transcriptomics and Metabolomics, Harvest periods

## Abstract

**Background:**

*Astragalus membranaceus* var. *mongholicus* (Astragalus), acknowledged as a pivotal “One Root of Medicine and Food”, boasts dual applications in both culinary and medicinal domains. The growth and metabolite accumulation of medicinal roots during the harvest period is intricately regulated by a transcriptional regulatory network. One key challenge is to accurately pinpoint the harvest date during the transition from conventional yield content of medicinal materials to high and to identify the core regulators governing such a critical transition. To solve this problem, we performed a correlation analysis of phenotypic, transcriptome, and metabolome dynamics during the harvesting of Astragalus roots.

**Results:**

First, our analysis identified stage-specific expression patterns for a significant proportion of the Astragalus root genes and unraveled the chronology of events that happen at the early and later stages of root harvest. Then, the results showed that different root developmental stages can be depicted by co-expressed genes of Astragalus. Moreover, we identified the key components and transcriptional regulation processes that determine root development during harvest. Furthermore, through correlating phenotypes, transcriptomes, and metabolomes at different harvesting periods, period D (Nov.6) was identified as the critical period of yield and flavonoid content increase, which is consistent with morphological and metabolic changes. In particular, we identified a flavonoid biosynthesis metabolite, isoliquiritigenin, as a core regulator of the synthesis of associated secondary metabolites in Astragalus. Further analyses and experiments showed that *HMGCR*, *4CL*, *CHS*, and *SQLE*, along with its associated differentially expressed genes, induced conversion of metabolism processes, including the biosynthesis of isoflavones and triterpenoid saponins substances, thus leading to the transition to higher medicinal materials yield and active ingredient content.

**Conclusions:**

The findings of this work will clarify the differences in the biosynthetic mechanism of astragaloside IV and calycosin 7-O-β-D-glucopyranoside accumulation between the four harvesting periods, which will guide the harvesting and production of Astragalus.

**Supplementary Information:**

The online version contains supplementary material available at 10.1186/s12870-024-05030-7.

## Introduction

*Astragalus membranaceus* var. *mongholicus* (Astragalus) is a widely used plant in today’s medical industry, which belongs to the legume family [1]. The widely grown herb is high in pharmacological active ingredient content and is suitable for multiple pharmaceutical food and industrial uses [2, 3]. Astragalus is one of the most commonly used traditional Chinese herbs and has been used for colorectal cancer, anti-aging, and antioxidation [4–6]. In addition, the dried roots of the plant have been added to foods to prevent diseases and maintain health in daily life [7]. The medicinal root, the carbohydrate storage organ that is also used for vegetative propagation, is the most economically important part of the Astragalus and provides an excellent model for studying organogenesis and evolution. Therefore, much of the research on Astragalus focuses on pharmacological effects and cultivation methods.

**Fig. 1:**
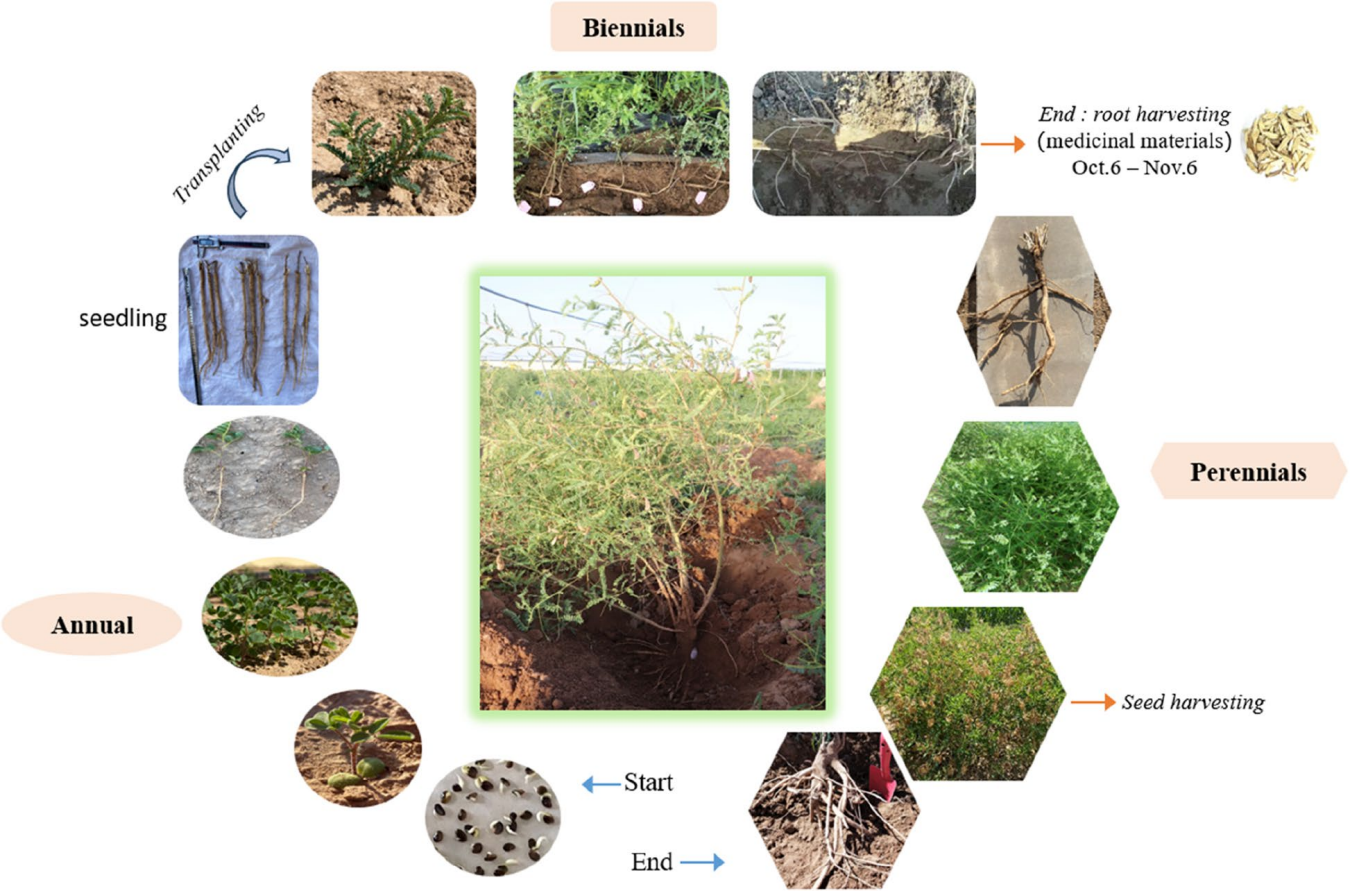
The important phenological growth stages in the whole growth process of perennial plant Astragalus

The roots of Astragalus grow vertically, and the length of the root can reach up to 80–100 cm. Astragalus is primarily distributed in Inner Mongolia, Gansu Province, Heilongjiang, and Shanxi in China, with a small amount of distribution in Russia, Korea, and Mongolia. These plants primarily grow on sandy loam and have good adaptability to arid and cold environments [8]. The growth period of Astragalus is at least 2 years but can reach 6 or more years, including beyond a dozen years. Two-year-old Astragalus is usually used as medicine and sold as commodities on the Chinese medicine market, while Astragalus growing for more than two years is mainly used to harvest seeds (Fig. 1). Previous research has focused on the difference between traditional medicinal pharmacological effects and plant phenotypes in Astragalus. However, the biosynthetic mechanisms of secondary metabolites accumulation in the roots of Astragalus at harvest remains to be clarified. Our study aimed to clarify the differences between the differences in root growth and development between 2-year-old plants at different harvest stages and explore the changes in astragaloside IV and calycosin 7-O-β-D-glucopyranoside in Astragalus during the harvest period.

Gene regulation is a generic term for a series of regulatory processes in plants, with specific expression of genes in different tissues and at different growth stages [9–11]. Gene regulation is a focal point of biological research, and the use of constructed regulatory networks can lead to new hypotheses and improved experimental designs, helping biologists to further analyze the regulatory mechanisms of biological processes such as development and adaptation to adversity [12]. Association analysis helps to identify important genes that respond to biological processes. For non-model species with a weak research foundation and a lack of functional genomics resources, association analysis of phenotypes and gene regulation processes can form a co-expression regulatory network between interactively expressed genes and co-expressed genes in a highly reliable regulatory network [13]. In addition to identifying gene interactions, gene regulatory network construction can also be used to explore the functions and biological patterns of genes from the network [14]. Such as, during strawberry receptacle development, two homologous genes LOST MERISTEM and WUSCHEL of strawberry play a key regulatory role in the development of receptacles, which are closely related to their growth and development [15]. Prediction of gene function based on association analysis can help to further improve the rate of gene function annotation, especially for species with weak research foundations and less functional annotation. Integrating gene annotation information into the process of metabolite synthesis can effectively excavate the real regulatory relationship among genes [16–18]. Gene and metabolite regulation can also be combined with phenotypic analysis to further explore the role of regulatory processes in phenotypic alterations [19, 20]. By speculating the regulatory relationship among phenotypes, genes, and metabolites at different harvest periods, it is possible to predict the function of genes, find key hub genes, and study the evolution between species. Combining phenotype and metabolite regulation processes for analysis has important application value in data mining of gene expression differences.

The growth and metabolite accumulation of medicinal roots during the harvest period is intricately regulated by a transcriptional regulatory network. One key challenge is to accurately pinpoint the harvest date during the transition from conventional yield content of medicinal materials to high and to identify the core regulators governing such a critical transition. To date, the mechanism of growth and development and metabolite biosynthesis in the harvest period of Astragalus has not been explored. Metabolomics and transcriptomics reflect the process of information passing from genes to metabolites in plants from multiple aspects. Combined analysis of phenotype and metabolomics was used to explore whether the metabolomic differences in different harvest periods of Astragalus were consistent with the changing trend of relevant genes. Thus, we measured the differences in root phenotypic characteristics between different harvest periods and then determined the contents of astragaloside IV and calycosin 7-O-β-D-glucopyranoside between different harvest periods by high-performance liquid chromatography (HPLC). Transcriptome sequencing and non-targeted metabolomics were performed on roots at different stages. Moreover, quantitative real-time polymerase chain reaction (qRT–PCR) was conducted to validate the conclusions drawn from transcriptomics. Finally, integrated analysis, Pearson correlation, and Short Time-series Expression Miner (STEM) analysis of the gene expression and metabolite data were also used to provide a comprehensive understanding of the biosynthetic mechanisms of root metabolites in different harvest periods.

The findings of this work will clarify the biosynthetic mechanism of two main pharmacodynamic components accumulation between different harvest periods, which will guide the rational harvest of Astragalus and provide an information-rich resource for investigating the functions of key components. Here, based on our gene expression profiles of Astragalus roots at different harvesting stages, we studied the molecular mechanisms of root development from the perspectives of phenotype and omics identified the optimal harvesting date of medicinal materials.

## Materials and methods

### Plant materials

*Astragalus membranaceus* (Fisch) Bge.var. *mongholicus* (Bge) Hsiao (Astragalus) seedlings were cultivated in mid-April in the Plantation for Inner Mongolia Agricultural University, Hohhot, China. The population of Astragalus seedlings were planted with a density of 20 cm × 15 cm (length by width), and the plot area 100 × 100 m. Astragalus grew up under sandy loam without fertilizer on rainfall conditions for 24 weeks. Medicinal roots at four periods (period A, Oct.6; period B, Oct.16; period C, Oct.26; period D, Nov.6 respectively) were collected in early October to cover the entire medicinal materials root harvesting processes (Fig. 1) [21]. Roots in each developmental stage measured for thirty independent biological replicates. These materials were separated into two parts: one part was rapidly frozen in liquid nitrogen and subsequently stored at -80℃ for RNA isolation and metabolome detection; another part was used for the analysis of astragaloside IV and calycosin 7-O-β-D-glucopyranoside content.

### Phenotypic characterization and quality identification of roots

The phenotypes of the Astragalus roots were studied under field conditions in the plantation. We recorded the field characteristics of roots at different developmental stages and the average root length, root diameter, root fresh weight, and thick lateral root of each stage thirty plants.

Astragaloside IV and calycosin 7-O-β-D-glucopyranoside content were analyzed as previously described [22, 23]. We accurately weighed 2.0 g dried samples and put them into 50 ml tubes, respectively. Then added 50 ml of methanol (80%, Contains 4% concentrated ammonia test solution). After shaking and mixing thoroughly, these samples were heating refluxed at 95℃ for 80 min, then evaporated to dryness, dissolved in 80% chromatographic methanol, and diluted to 10 ml volumetric flask. Filtered with 0.22 μm organic filter membrane, and then we transferred 1 ml of the liquid into the sample bottle to analyze the content of each astragaloside IV using HPLC. The HPLC column (Thenmo-C18; 100 mm × 2.1 mm, 1.9 μm; Hypersil GOLD aO) was used with an evaporative Light-scattering detector. We used 70% acetonitrile as the mobile phase; the flow velocity was 0.2 ml·min^−1^ and the column temperature was 30℃. The retention times of standards were used to identify each content was obtained based on the external standard curve. Data from at least six technical replicates are presented as the mean. Furthermore, We accurately weighed 2.0 g dried samples put them into 50 ml tubes, and then added 50 ml of methanol. After shaking and mixing thoroughly, the ultrasound was placed in a 40 ℃ ultrasound instrument for 120 min. The HPLC was used with a UV detector. We used 70% acetonitrile as the mobile phase; the flow velocity was 0.2 ml·min^−1^ and the column temperature was 30℃. Other processes and analyses were the same as described above.

### RNA extraction and root transcriptome analysis

For RNA-seq analysis, total RNA was extracted from the roots (peard A - D) using TRIzol® Reagen with the protocol provided by the manufacturer. Then RNA quality was determined by 5300 Bioanalyser (Agilent) and quantified using the ND-2000 (NanoDrop Technologies). Only high-quality RNA sample (OD260/280 = 1.8 ~ 2.2, OD260/230 ≥ 2.0, RIN ≥ 6.5, 28 S: 18 S ≥ 1.0, > 1 µg) was used to construct sequencing library. RNA purification, reverse transcription, library construction, and sequencing were performed at Shanghai Majorbio Bio-pharm Biotechnology Co., Ltd. (Shanghai, China) according to the manufacturer’s instructions (Illumina, San Diego, CA). The astragalus roots RNA-seq transcriptome library was prepared following Illumina® Stranded mRNA Prep, Ligation from Illumina (San Diego, CA) using 1 µg of total RNA (four samples in three biological replicates). Shortly, messenger RNA was isolated according to polyA selection method by oligo(dT) beads and then fragmented by fragmentation buffer firstly. Secondly, double-stranded cDNA was synthesized using a SuperScript double-stranded cDNA synthesis kit (Invitrogen, CA) with random hexamer primers (Illumina). Then the synthesized cDNA was subjected to end-repair, phosphorylation and ‘A’ base addition according to Illumina’s library construction protocol. Libraries were size selected for cDNA target fragments of 300 bp on 2% Low Range Ultra Agarose followed by PCR amplified using Phusion DNA polymerase (NEB) for 15 PCR cycles. After quantified by Qubit 4.0, paired-end RNA-seq sequencing library was sequenced with the NovaSeq 6000 sequencer (2 × 150 bp read length).

The raw paired end reads were trimmed and quality controlled by fastp (https://github.com/OpenGene/fastp) [24] with default parameters. Then clean data from the samples (Astragalus) were used to do de novo assembly with Trinity (https://github.com/trinityrnaseq/trinityrnaseq/wiki) [25]. To increase the assembly quality, all the assembled sequencs were filtered by CD-Hit and transrate. The assembled transcripts were searched against the NCBI protein nonredundant (NR), COG, and KEGG databases using Diamond to identify the proteins that had the highest sequence similarity with the given transcripts to retrieve their function annotations and a typical cut-off E-values less than 1.0 × 10^−5^ was set. BLAST2GO [26] program was used to get GO annotations of unique assembled transcripts for describing biological processes, molecular functions and cellular components. Metabolic pathway analysis was performed using the Kyoto Encyclopedia of Genes and Genomes (KEGG) [27].

The TPM values of each astragalus root gene were obtained by RSEM (Version 1.3.1, http://deweylab.github.io/RSEM/) using the mapped output. Two R packages, i.e. CORRPLT and PRCOMP, were used to perform hierarchical clustering and PCA analysis, respectively. Gene expression level was calculated using transcripts per million reads (TPM). Differentially expressed genes (DEGs) were screened by DESeq2 (http://bioconductor.org/packages/stats/bioc/DESeq2/) with a filter criteria of fold change (FC) > 2.0 and q-value < 0.05 [11, 28]. Gene expression patterm analysis was perform by Short Time-series Expression Miner software(STEM) [29] on the Majorbio Cloud Platform, a free online platform for data analysis (www.omicshare.com/tools). Maximum Unit Change in model profiles between time points is 1. Maximum outmput profiles number is 20 (similar profiles will be merged). Minimum ratio of fold change of DEGs is no less than 2.0. Gene Ontology database (GO) and Kyoto Encyclopedia of Genes and Genomes (KEGG) database were utilized to annotate which DEGs were significantly enriched at Benjamini and Hochberg (BH)- corrected false discovery rate < 0.05 by Goatools (Version 0.6.5, https://github.com/tanghaibao/GOatools) and KOBAS (Version 2.1.1, http://kobas.cbi.pku.edu.cn/home.do) [30, 31]. The test for over-representation of Gene Ontology (GO) terms within differentially expressed genes (FDR < 0.05, |LogFC| > 1) was conducted using edgeR’s in-built goana function with FDR cut-off set at 0.05, taking into account gene length bias. Log (base 2)-fold changes resulting after the linear model fit with edgeR were used as per gene score, and a two-sample t-test was run to compare mean log-fold changes per set relative to mean background log-fold change calculated using all filtered genes.

### Untargeted root metabolome analysis

50 mg samples were accurately weighed, and the metabolites were extracted using a 400 µL methanol : water (4:1, v/v) solution with 0.02 mg/mL l-2-chlorophenylalanin as internal standard (four samples in six biological replicates). The mixture was allowed to settle at -10℃ and treated by High throughput tissue crusher Wonbio-96c( Shanghai Wanbo Biotechnology Co., LTD) at 50 Hz for 6 min, then followed by ultrasound at 40 kHz for 30 min at 5℃. The samples were placed at -20 ℃ for 30 min to precipitate proteins. After centrifugation at 13,000 g at 4 ℃ for 15 min, the supernatant was carefully transferred to sample vials for LC-MS/MS analysis. Pooled quality control (QC) sample was prepared by mixing equal volumes of each extracted supernatant and injected at regular intervals (every 8 samples). The instrument platform for LC-MS analysis is UHPLC-Q Exactive system of Thermo Fisherm Scientific.

2µL of sample was separated by HSS T3 column (100 mm × 2.1 mm i.d., 1.8 μm) and then entered into mass spectrometry detection. Flow rate was 0.4 mL/min, and column temperature was 40 ℃. Mobile phases consisted of 0.1% formic acid in water : acetonitrile (95:5, v/v) (solvent A) and 0.1% formic acid in acetonitrile : isopropanol : water (47.5:47.5:5, v/v) (solvent B). Solvent gradient changed according to the following conditions: 0% B to 5% B (0–0.1 min); 5% B to 25% B (0.1–2 min); 25% B to 100% B (2–9 min); 100% B to 100% B (9–13 min); 100% B to 0% B (13–13.1 min); 0% B to 0% B (13.1–16 min) for equilibrating the systems. The mass spectrometric data was collected using a Thermo UHPLC-Q Exactive Mass Spectrometer equipped with an electrospray ionization (ESI) source. The optimal conditions were set as followed: heater temperature, 400 ℃; Capillary temperature, 320 ℃; sheath gas flow rate, 40 arb; Aux gas flow rate, 10 arb; ion-spray voltage floating (ISVF), -2800 V in negative mode and 3500 V in positive mode, respectively; Normalized collision energy, 20-40-60 V rolling for MS/MS. Full MS resolution was 70,000, and MS/MS resolution was 17,500. Data acquisition was performed with the Data Dependent Acquisition (DDA) mode, and detected mass range of m/z 50 to 1000.

After the mass spectrometry detection is completed, the raw data of LC/MS is preprocessed by Progenesis QI (Waters Corporation, Milford, USA) software, and a three-dimensional data matrix in CSV format is exported. The information in this three-dimensional matrix includes: sample information, metabolite name and mass spectral response intensity. Internal standard peaks, as well as any known false positive peaks (including noise, column bleed, and derivatized reagent peaks), were removed from the data matrix, deredundant and peak pooled. At the same time, the metabolites were searched and identified, and the main database was the HMDB(http://www.hmdb.ca/), Metlin( https://metlin.scripps.edu/) and Majorbio Database. The data after the database search is uploaded to the Majorbio cloud platform (https://cloud.majorbio.com) for data analysis.Metabolic features detected at least 80% in any set of samples were retained. After filtering, minimum metabolite values were imputed for specific samples in which the metabolite levels fell below the lower limit of quantitation and each Metabolic features were normalized by sum. In order to reduce the errors caused by sample preparation and instrument instability, the response intensity of the sample mass spectrum peaks was normalized by the sum normalization method, and the normalized data matrix was obtained. At the same time, variables with relative standard deviation (RSD) > 30% of QC samples were removed, and log10 logarithmization was performed to obtain the final data matrix for subsequent analysis.

Processed data was treated with a log-transformed processing and identified based on HMDB, Metlin, and Majorbio database. Perform variance analysis on the matrix file after data preprocessing. The R package ropls (Version 1.6.2) performed principal component analysis (PCA) and orthogonal least partial squares discriminant analysis (OPLS-DA), and used 7-cycle interactive validation to evaluate the stability of the model. In addition, student’s t-test and fold difference analysis were performed. The selection of significantly different metabolites was determined based on the Variable importance in the projeciton (VIP) obtained by the OPLS-DA model and the p-value of student’s t test, and the metabolites with VIP > 1, *p* < 0.05 were significantly different metabolites. Enrichment of KEGG pathway was carried out by Goatools and KOBAS 2.1.1. Integrated transcriptome and metabolome analysis were conducted using iPath 3.0 to profile significantly altered metabolic pathways. Differential metabolites among two groups were summarized, and mapped into their biochemical pathways through metabolic enrichment and pathway analysis based on database search (KEGG, http://www.genome.jp/kegg/). These metabolites can be classified according to the pathways they involved or the functions they performed. Enrichment analysis was usually to analyze a group of metabolites in a function node whether appears or not. The principle was that the annotation analysis of a single metabolite develops into an annotation analysis of a group of metabolites. scipy.stats (Python packages) ( https://docs.scipy.org/doc/scipy/ ) was exploited to identify statistically significantly enriched pathway using Fisher’s exact test.

### qRT-PCR of key gene expression

qRT-PCR was used to determine the expression of genes related to astragaloside IV and calycosin 7-O-β-D-glucopyranoside synthesis. Total root RNA was isolated from three biological replicate samples of each group by QIAzolLysisReagent (Qiagen, Germany). Reverse transcription and polymerase chain reaction were conducted by PrimeScript™ 1st Strand cDNA Synthesis Kit and SYBR®Premix Ex Taq™ II(Tli RNaseH Plus)(Takara Biomedical Technology (Beijing) Co., Ltd., China). Targeted gene was measured using Heal Force RealTime PCR System (Heal Force CG-05, Hangzhou Jingle Scientific Instrument Co., Ltd., China) as described previously, and each sample was measured for three times technical replicates. The reaction system was 20 µL, including 10 µL of Tli RNaseH Plus, 0.4 µL of Primer F (10 µM), 0.4 µL of Primer R (10 µM), 2 µL of cDNA, ROX Reference Dye 0.08µL and 7.12 µL of ddH_2_O. All reactions were conducted in 96-well plates. The qPCR protocol included annealing at 95 ℃ for 60 s, followed by 40 cycles of 95 ℃ for 30 s, primer annealing at 60 ℃ for 30 s, and extension at 72 ℃ for 20 s. A negative control without template for each primer pair was included in each qPCR analysis. Expression was normalized to GAPDH and calculated using 2^−△△Ct^ [32]. NCBI Primer-BLAST was used to design the gene-specific primers. Primer sequences are listed in Table S17.

### Statistical analysis

Data was processed statistically by analysis of variance (ANOVA), and detected significant differences among mean values at *P* < 0.05 using the least significance difference (LSD) test through SPSS 23.0 software (IBM, https://www.ibm.com). An alpha value of *P* < 0.05 was considered statistically significant. RSEM, GOATOOLS, and KOBAS were adopted to RNA-seq data. The ROPLS (Version 1.6.2) R package from Bioconductor on Majorbio Cloud Platform were used to conduct a multivariate statistical analysis. The figures were prepared using Origin 9.0 and Excel 2019 software. Details of the data analysis are described in support file.

## Results

### Identification of root morphological indexes and analysis of medicinal materials quality

**Fig. 2:**
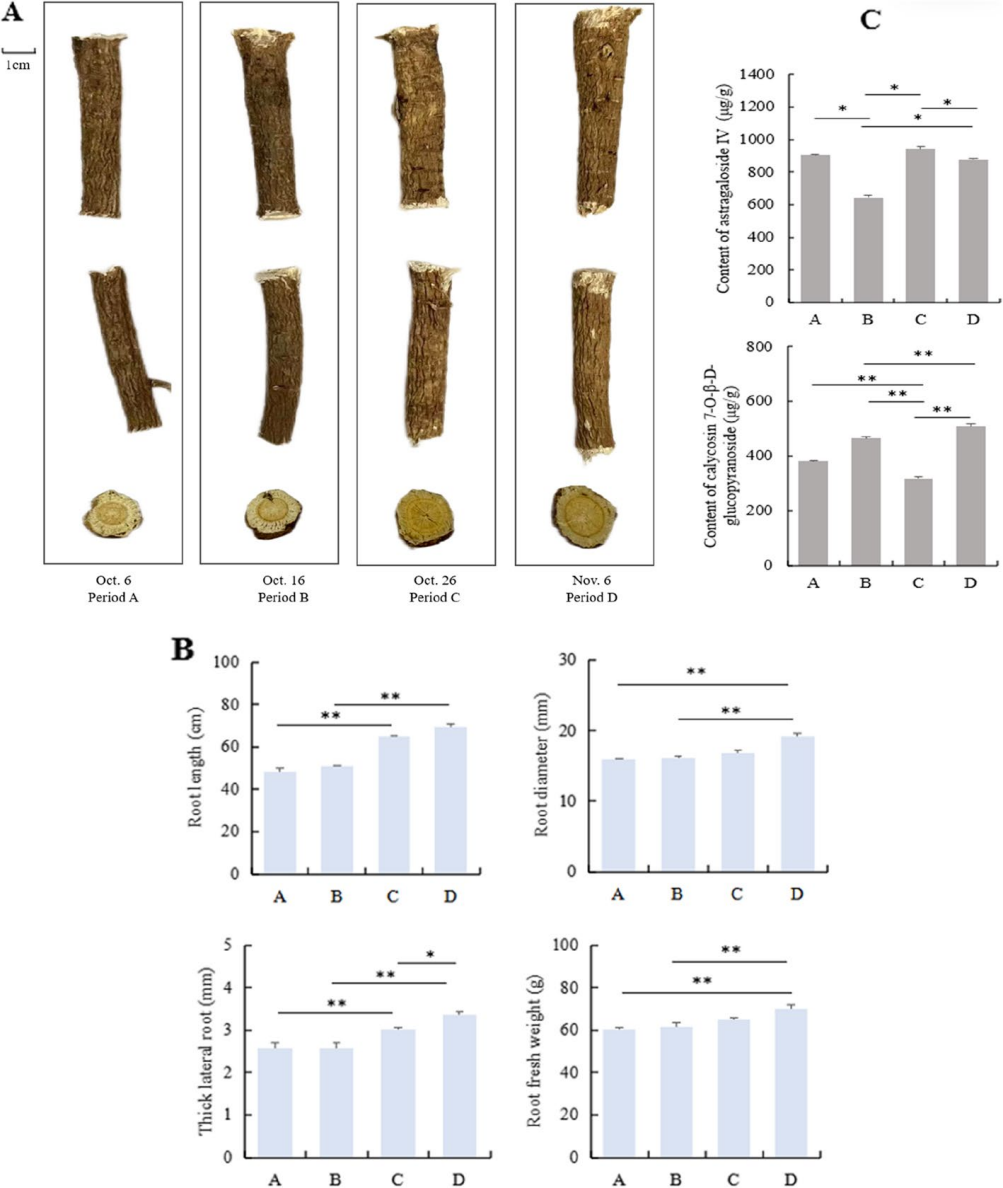
Morphological changes of roots and quality of medicinal materials in different harvesting periods of Astragalus. **A** Morphology of Astragalus roots at different developmental stages. Period A (harvested on October 10); Period B (harvested on October 20); Period C (harvested on October 30); Period D (harvested on November 10). **B** Differential values of phenotypic trait indicators at different harvesting periods. **C** Variation in astragaloside IV and calycosin 7-O-β-D-glucopyranoside contents of Astragalus roots at different developmental stages. Error bars indicate standard errors (*n* = 30). * and ** indicate significant difference compared at *P* < 0.05 and *P* < 0.01, respectively

Given the different harvest times, some different physical characteristics have also emerged in the root of Astragalus, such as the length and diameter of the principal roots and the apparent color. Several important phenotypes and main active ingredient parameters were analyzed in roots at four developmental stages (period A - D) representing major events occurring within the harvest period of Astragalus medicinal materials. The growth of Astragalus root was more promoted in solutions with delay of harvest time (Fig. 2A). The diameter of the main root was finer at 1–5 cm and 10–15 cm at the beginning of harvest (periods A and B). Xylem phloem showed that the phloem color was lighter in the early stage of harvesting ( periods A and B ), and the phloem color was darker in the later stage of harvesting. The root epidermis color showed that the root epidermis color was darker in the early harvest period ( periods A and B ), and the root epidermis color was lighter in the late harvest period (Fig. 2A). Compared with the A and B stages, the root length, root diameter, root fresh weight and thick lateral root of all plants increased significantly with the delay of the harvest period. Under delayed harvest, root length of Astragalus root growth parameters ranged from 48.38 to 69.45 cm, root diameter ranged from 15.94 to 19.24 mm, and thick lateral root ranged from 2.56 to 3.37 mm, whereas root fresh weight ranged from 60.52 to 70.23 g. The increase of root biomass in period D was significantly higher than that in the other three periods (*P* < 0.05, *P* < 0.01). The increase of root biomass in C stage was significantly higher than that in A and B stages (*P* < 0.05, *P* < 0.01). The root length value showed similar trends to thick lateral root, and root diameter value showed similar trends to root fresh weight (Fig. 2B). Overall, root phenotype change was evident with the delay of the harvest period. The root fresh weight of the period D being 1.16 times greater than that with period A.

Contents of two main active components in Astragalus roots at different harvest periods were determined. Astragaloside IV and calycosin 7-O-β-D-glucopyranoside content in roots changed significantly with the delay of harvest time. The concentration of astragaloside IV ranged from 646 to 942 µg/g, and the concentration of calycosin 7-O-β-D-glucopyranoside ranged from 318 to 509 µg/g. The value of period C of astragaloside IV content was larger and period B was smaller than other harvesting times, and period D of calycosin 7-O-β-D-glucopyranoside content was larger and period C was smaller than other harvesting time, indicating that the astragaloside IV and calycosin 7-O-β-D-glucopyranoside content of Astragalus was more sensitive to the harvest period. Overall, root morphology changed was evident with the delay of harvest time. This doubtlessly promoted the accumulation mode of the two active ingredients, with the content of astragaloside IV of the period C being 1.5 times greater than that with period B, and the calycosin 7-O-β-D-glucopyranoside content in period D was 1.6 times that in period C (Fig. 2C).

### Effects of harvesting period on Astragalus roots transcriptome

**Fig. 3:**
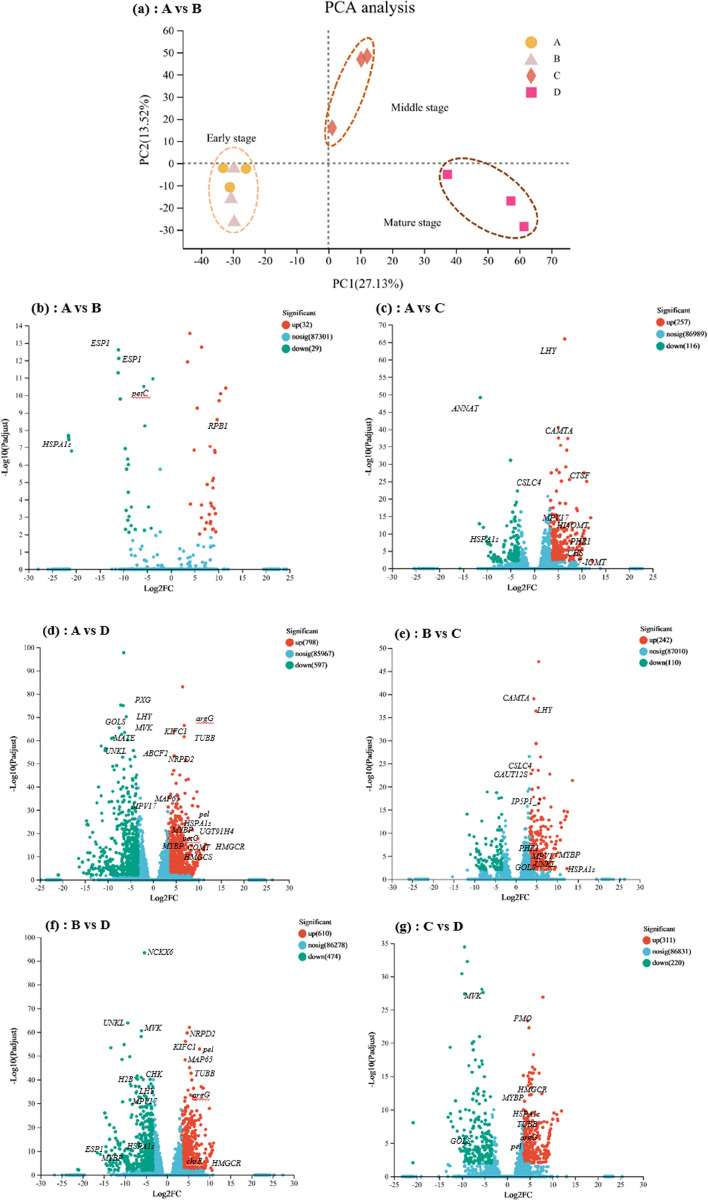
Effects of harvesting periods on roots transcriptome. **a** Principal component analysis (PCA) plot showing the clustering of transcriptomes of Astragalus roots at different developmental stages. Volcano plot of significant DEGs of stage A vs. B (**b**), stage A vs. C (**C**), stage A vs. D (**D**), stage B vs. C (**e**), stage B vs. D (**f**), and stage C vs. D (**g**). Red dots, upregulated; green dots, downregulated

RNA Seq analysis was applied to root samples from A, B, C, and D groups. A total of 42.64–58.42 million clean reads were obtained in each sample after quality checks (Table S1). PCA analysis revealed that the harvesting period divided the Astragalus root transcriptome into three groups: (i) early stage (A and B); (ii) middle stage (C); (iii) mature stage (D) (Fig. 3a), which reflects the progression from fibrous roots to mature(Fig. 1a). Stage D showed clear differences compared to A (Fig. S1c). PC1 filtering separated stages C and A, C and B, D and B, D and C, which accounted for 37.17%, 37.26%, 38.17%, and 39.26% of total variation, respectively (Fig. S1b, d, e & f). However, slight shifts were observed in stage B compared to A, indicating that they might be at a critical stage before the swelling transition to mature stage.

Differential expression analysis showed that 1395 DEGs were identified in stage D compared to A, of which 597 and 798 genes were downregulated and upregulated, respectively (Fig. 3d, see also Table S2). Relative to stage A, 373 DEGs were detected in stage C (Fig. 3c) and only 61 DEGs were identified in B, indicating that harvesting period had a lesser effect on mid-harvesting transcription (Fig. 3b, Fig. S1a). Harvesting period also produces 1084 DEGs in stage D relative to stage B (Fig. 3f).

**Fig. 4:**
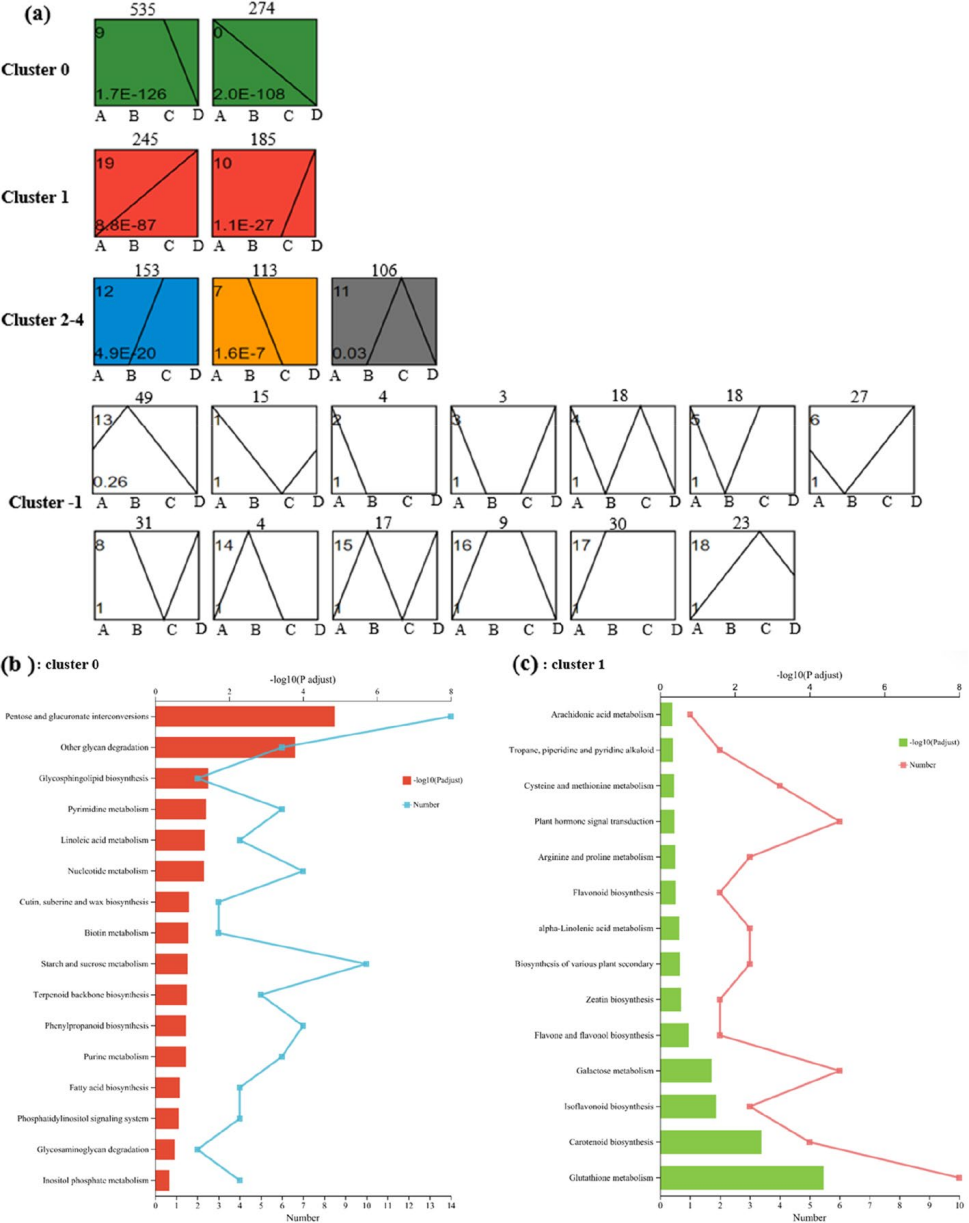
(**a**) Patterns of significant differentially expressed genes (DEGs) expressions in Astragalus roots across harvesting periods inferred by Short Time-series Expression Miner (STEM) analysis. The STEM (Version 1.3.11) program classified the 2167 significant expression genes into six main possible model clusters (Supplementary Table 3) according to the temporal gene expression patterns, and within these recognized 20 significant patterns of gene expression (*p* < 0.05). Each profile corresponds to a rectangle, the number in the upper left corner of the rectangle is the ID of the profile, the line is the trend of the expression over time, and the value in the lower left corner is its corresponding significance level P-value. In each frame, the different colors represent the expression tendency of all the DEGs; the number of DEGs belonging to each pattern was labeled above the frame. Where, (1) trend graph with colors: profiles with the same colour represent the same cluster (profiles with similar trends are grouped together); (2) trend graph without colors: the pattern of the profile is a statistically non-significant trend. Enriched Kyoto encyclopedia of genes and genomes (KEGG) pathways (metabolism) of cluster 0 (**b**) and 1 (**c**) that were significantly overrepresented for differentially expressed genes in Astragalus roots of different harvest periods

Through Short Time-series Expression Miner analysis, the significant expression DEGs at different harvesting periods were clustered into twenty profiles (from profile 0 to 19) based on the expression patterns of genes (Fig. 4a, Table. S3) to obtain DEGs that change significantly with the period of harvest. The most represented clusters were profiles 0 and 19 (*P* < 0.05). In profile 0, the expressions of 274 genes significantly decreased with the extension of harvesting periods, and in profile 19, the expression of 245 genes were increased with extending harvesting periods (*P* < 0.05), accounting for 27.92% of all significant expression DEGs.

To further understand the changes in transcription, KEGG enrichment analysis was implemented for the genes belonging to clusters 0 and 1 (Fig. 4b and c). The DEGs of clusters 0 and 1 were assigned into 74 and 53 KEGG pathways in all the five KEGG categories, respectively, including metabolism, genetic information processing, environmental information processing, cellular processes, and organismal systems. Among them, metabolism accounted for most, at 79.73% for cluster 0, and 81.13% for cluster 1. The significantly enriched KEGG pathways related to metabolism included carbohydrate metabolism, glycan biosynthesis, and nucleotide metabolism (up-regulated) (Fig. 4b, Table. S4) and responses to metabolism of other amino acids, metabolism of terpenoids and polyketides, and biosynthesis of other secondary metabolites (down-regulated) (Fig. 4c, Table. S4).

Combined with STEM and KEGG enrichment analysis, 31 genes (Table. S4) related to the accumulation of important secondary metabolites during harvest of Astragalus were identified in eight pathways related to terpenoid backbone biosynthesis (map00900), phenylpropanoid biosynthesis (map00940), biosynthesis of various plant secondary metabolites (map00999), brassinosteroid biosynthesis (map00905), zeatin biosynthesis (map00908), carotenoid biosynthesis (map00906), isoflavonoid biosynthesis (map00943) and flavonoid biosynthesis (map00941).

### Annotation of DEGs between harvest periods a and B vs. D

**Fig. 5:**
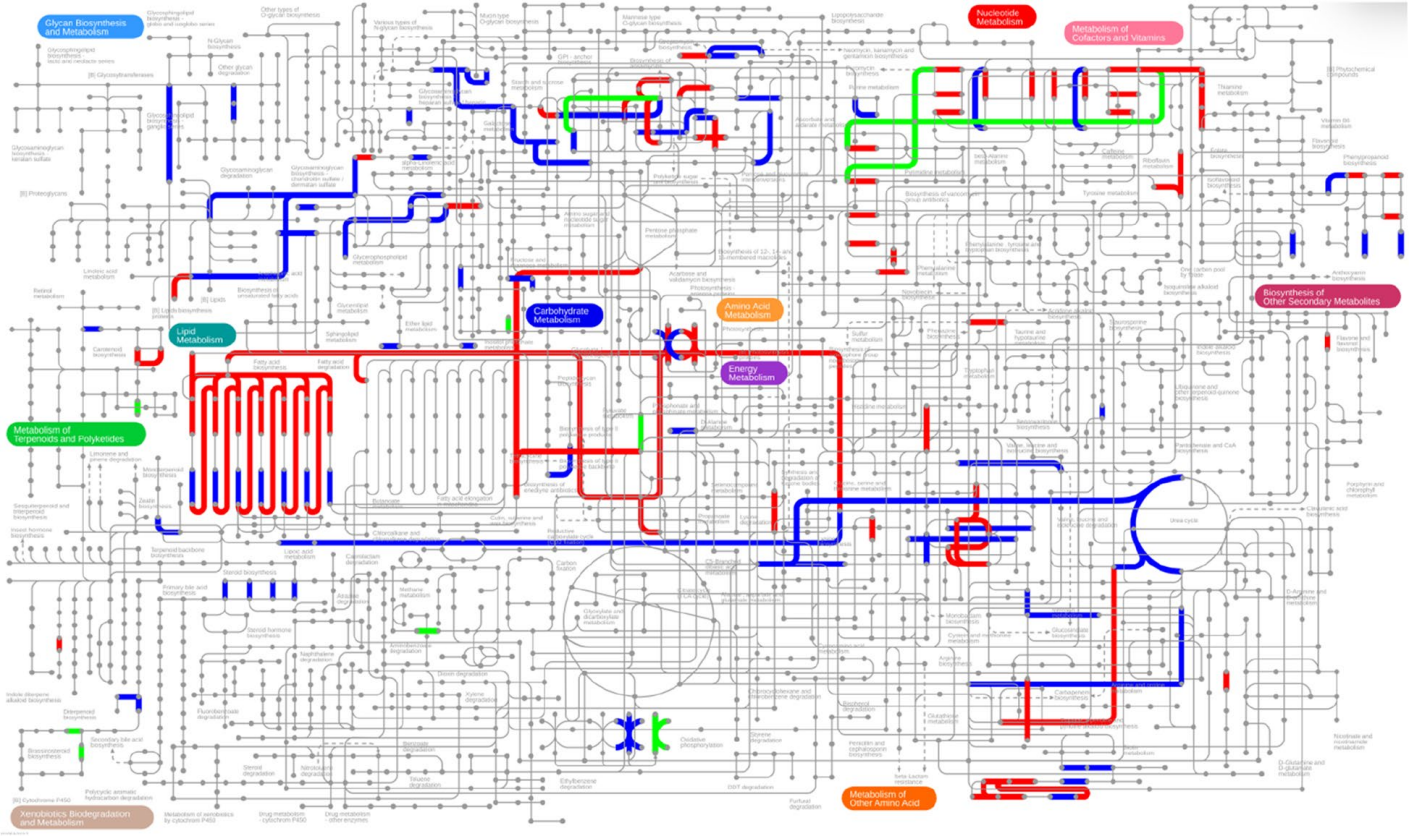
Integrated transcriptome and metabolome analysis in iPath 3.0 for visualizing KEGG general metabolic pathway map. Dots and lines represent differential metabolites and DEGs, respectively. Red, stage A vs. D; green, stage B vs. D; blue, shared metabolites and DEGs. These metabolic maps were visualized and analyzed by the iPath3.0 online tool (http://pathways.embl.de)

Since DEGs are significantly enriched between A vs. D and B vs. D, we further analyzed the global metabolic pathways of the DEGs via iPath3.0. As shown in Fig. 5, most DEGs were annotated to lipid metabolism, biosynthesis of other secondary metabolites, energy metabolism, glycan biosynthesis and metabolism, carbohydrate metabolism, nucleotide metabolism, metabolism of terpenoids and polyketides, and amino acid metabolism. Among these DEGs, terpenoid backbone biosynthesis (map00900, 6 unigenes), cutin, suberine and wax biosynthesis (map00073, 5 unigenes), and alanine, aspartate and glutamate metabolism (map00250, 2 unigenes) in metabolism of terpenoids and polyketides, lipid metabolism, and amino acid metabolism were significantly enriched, phenylpropanoid biosynthesis (map00940, 11 unigenes) and Isoflavonoid biosynthesis (map00943, 2 unigenes) in biosynthesis of other secondary metabolites were significantly enriched (Table. S5), indicating that the metabolism of terpenoids and polyketides, lipid metabolism, amino acid metabolism, and biosynthesis of other secondary metabolites of *Astragalus* roots under different harvest periods has a significant response, which may affect the synthesis of multiple secondary metabolites.

**Fig. 6:**
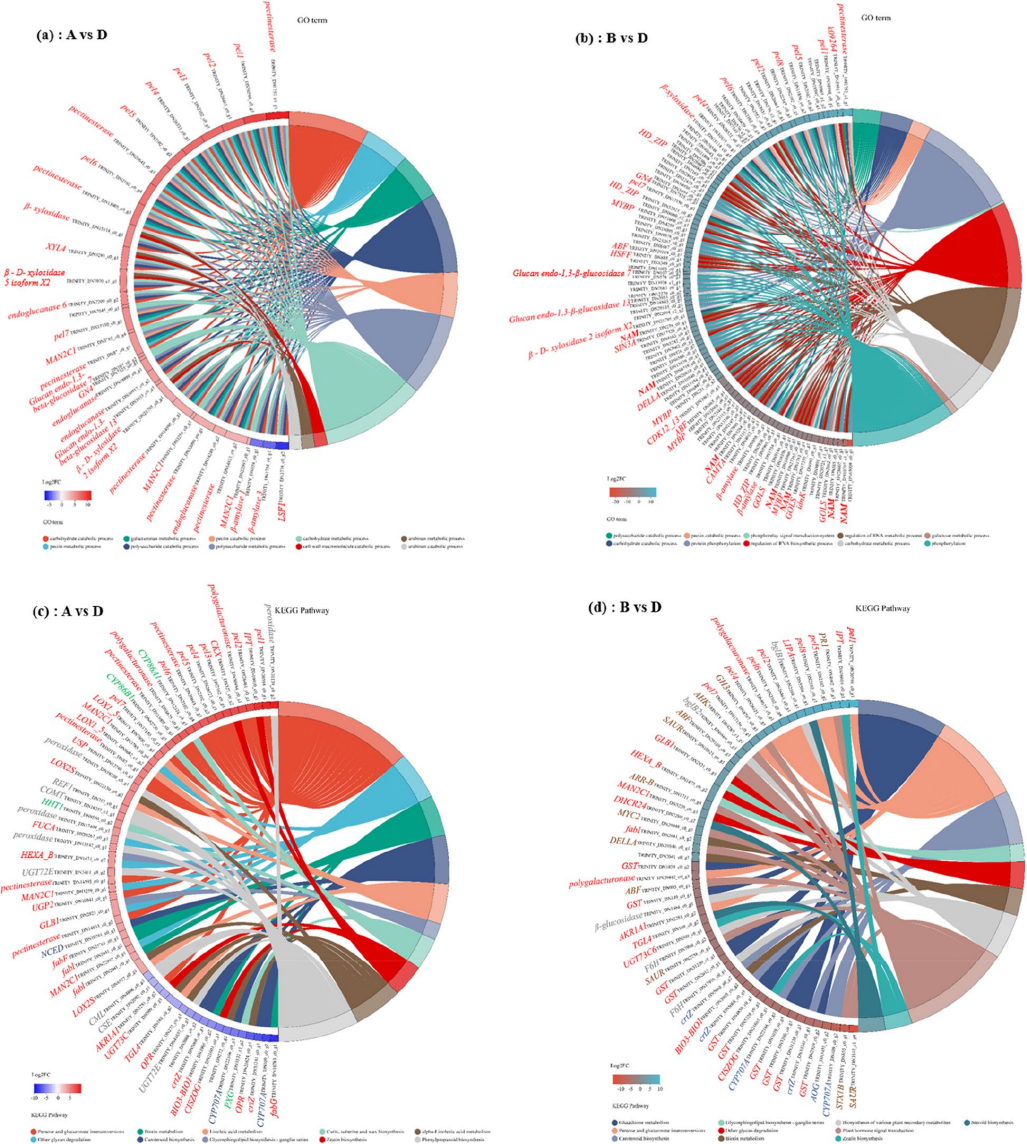
GO enrichment analysis. GO Chord plot of top 10 ranked overrepresented GO terms for different harvest periods Astragalus root. The genes are linked to their assigned terms via colored ribbons. Genes are ordered according to the observed log-fold change (log2FC), which is displayed in descending intensity of squares displayed next to the selected genes

From the 161 overrepresented GO terms (FDR < 0.05) associated with the 32 top DEGs for harvest period A vs. D, the top seven ranked were carbohydrate metabolic process, polysaccharide metabolic process, polysaccharide catabolic process, pectin metabolic process, galacturonan metabolic process, and pectin catabolic process (Fig. 6a and Table S6). The presence of starch and sucrose metabolism-related genes [glucan endo-1,3-beta-glucosidase 4 (*GN4*) and endoglucanase and glycosyl hydrolase family 14 (β-amylase 1 and 3)] was directly related to carbohydrate metabolic process. Several pectate lyase genes (pel1, pel2, pel3, pel4, pel5, pel6, and pel7), with a particularly pronounced downregulation in harvest period D, were also associated with other polysaccharide metabolic and catabolic, pectin metabolic and catabolic, and galacturonan metabolic-related sets. The remainder of the top five GO terms had many genes in common. In addition to *XYL4* and *GN4* glycosyl hydrolase family, interesting unigenes encompassed alpha-mannosidase (*MAN2C1*), involved in multiple processes such as other glycan degradation and carbohydrate transport and metabolism.

From the 215 overrepresented GO terms (FDR < 0.05) associated with the 98 top DEGs for harvest period B vs. D, the top five ranked were phosphorylation, protein phosphorylation, regulation of RNA biosynthetic process, regulation of RNA metabolic process, and carbohydrate metabolic process (Fig. 6b and Table S7). The polysaccharide catabolic process and pectin catabolic process were also found in the overrepresented GO terms list for the harvest period B vs. D. The largest set of no apical meristem (NAM) protein and *MYBP*, *HD-ZIP2*, *ABF*, *HSFF*, *SIN3A*, *DELLA*, and *CAMTA* genes was found in the regulation of RNA biosynthetic process. Some of the genes were associated only with the carbohydrate metabolic process term and posttranslational modification, protein turnover such as glucan endo-1,3-beta-glucosidase 4 (*GN4*), gluconokinase (*idnK*) and inositol 3-alpha-galactosyltransferase (*GOLS*). However, most of the genes were common to the top five GO terms. In addition to the omnipresent pel1 -pel7 pectinesterase, two other protein phosphorylation and polysaccharide catabolic process-related genes are worth mentioning; cyclin-dependent kinase 12/13 (*CDK12_13*) and β-amylase, the former known as the signal transduction mechanisms of the late plant growth.

**Fig. 7:**
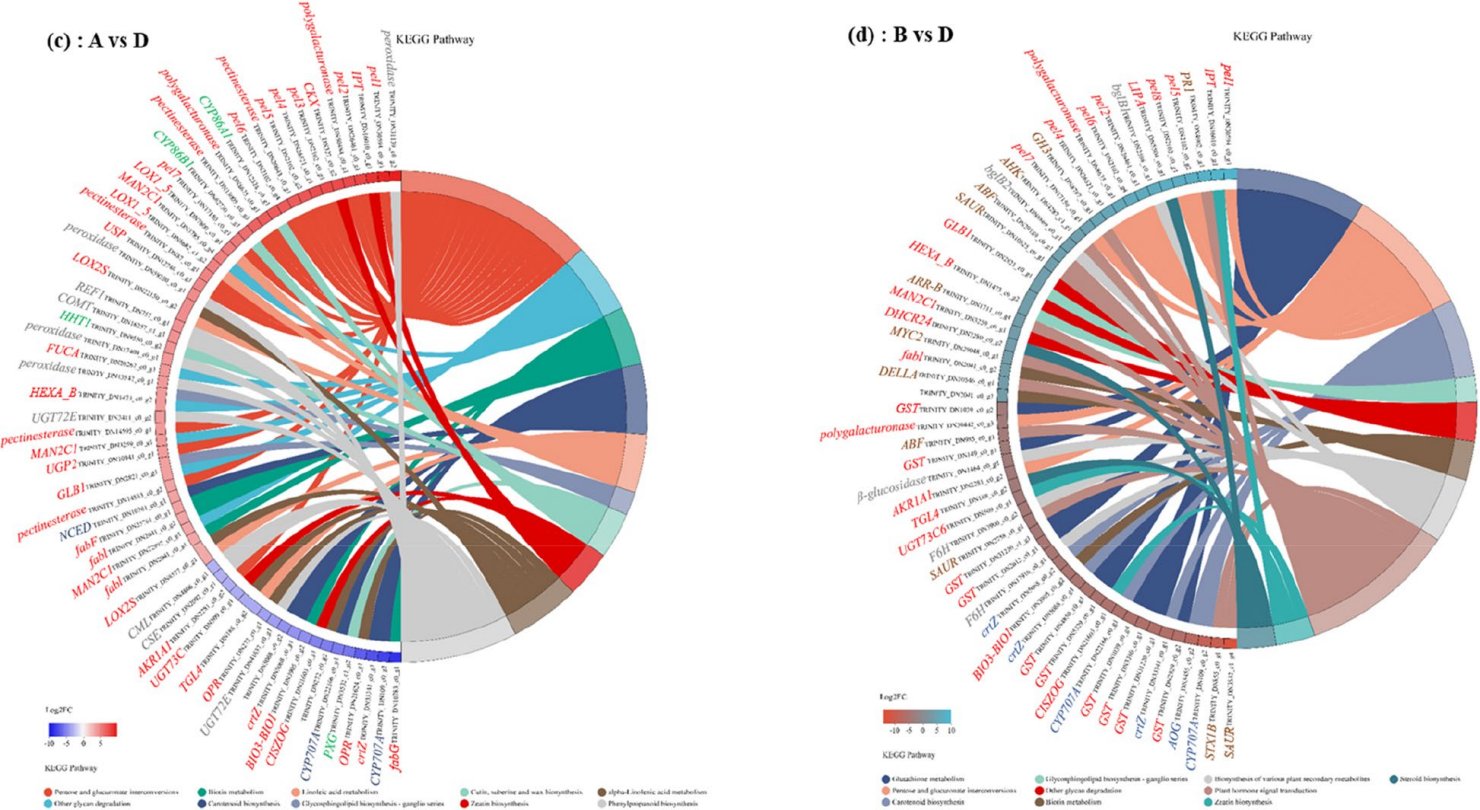
KEGG enrichment analysis. KEGG Chord plot of top 10 ranked overrepresented KEGG pathways for different harvest periods Astragalus root. The genes are linked to their assigned terms via colored ribbons. Genes are ordered according to the observed log-fold change (log2FC), which is displayed in descending intensity of squares displayed next to the selected genes

In Kyoto Encyclopedia of Genes and Genomes (KEGG) pathway enrichment analysis, 115 DEGs were annotated in the 14 most significant pathways, including phenylpropanoid biosynthesis, pentose and glucuronate interconversions, zeatin biosynthesis, cutin, suberine and wax biosynthesis, linoleic acid metabolism, other glycan degradation, alpha-Linolenic acid metabolism, glycosphingolipid biosynthesis - ganglio series, carotenoid biosynthesis, biotin metabolism, plant hormone signal transduction, steroid biosynthesis, biosynthesis of various plant secondary metabolites, and glutathione metabolism (Fig. 7).

From the 65 overrepresented KEGG pathways (FDR < 0.05) associated with the 60 top DEGs for harvest period A vs. D, the top five ranked were pentose and glucuronate interconversions, phenylpropanoid biosynthesis, cutin, suberine and wax biosynthesis, carotenoid biosynthesis, and alpha-Linolenic acid metabolism (Fig. 7a and Table S8). The presence of carbohydrate transport and metabolism-related genes peroxidase and some other functions [coniferyl-aldehyde dehydrogenase (*REF1*) and caffeic acid 3-O-methyltransferase (*COMT*) and coniferyl-alcohol glucosyltransferase (*UGT72E*), caffeoyl shikimate esterase (*CSE*)] was directly related to phenylpropanoid biosynthesis. Several cutin, suberine and wax biosynthesis-related genes (*CYP86A1*, *CYP86B1*, *HHT1*, and *PXG*), with a particularly pronounced upregulation or downregulation in harvest period D, were also associated with secondary metabolites biosynthesis and transport and catabolism. In addition to the *pel1* - *pel7* pectinesterase, interesting unigenes encompassed 9-cis-epoxy carotenoid dioxygenase (*NCED*) and abscisic acid 8’-hydroxylase (*CYP707A*) involved in carotenoid biosynthesis, which plays a role in Secondary metabolites biosynthesis and transport.

From the 57 overrepresented KEGG pathways (FDR < 0.05) associated with the 55 top DEGs for harvest period B vs. D, the top five ranked were plant hormone signal transduction, glutathione metabolism, pentose and glucuronate interconversions, carotenoid biosynthesis and biosynthesis of various plant secondary metabolites (Fig. 7b and Table S9). The presence of carbohydrate transport and metabolism-related genes beta-glucosidase (*bglB*) and some other functions [feruloyl-CoA 6-hydroxylase (*F6H*)] was directly related to biosynthesis of various plant secondary metabolites. Several cyanoamino acid metabolism genes (*bglB1* and *bglB2*), with a particularly pronounced downregulation in harvest period D, were also associated with other starch and sucrose metabolism. The largest set of *PR1*, *GH3*, *AHK*, *ABF*, *SAYR*, *ARR-B*, *MYC2*, *DELLA*, and *STX1B* genes was found in the regulation of plant hormone signal transduction. Some of the genes were associated with the induction and synthesis of Indole-3-acetic acids, such as auxin responsive *GH3* gene family (*GH3*), SAUR family protein (*SAUR*), which plays a role in *GH3* auxin-responsive promoter, and auxin responsive protein. Several secondary metabolites biosynthesis, transport, and catabolism genes (*crtZ*, *CYP707A*, *AOG*), with a particularly pronounced upregulation in harvest period D. These may be the key genes responsible for the significant reduction in secondary metabolite content at harvest D. Also, the *crtZ*, *AOG* and *CYP707A* genes may be key genes responsible for the marked change in harvest period D carotenoid biosynthesis.

Overall, GO and KEGG enrichment analysis was carried out to characterize significant functional DEGs caused by different harvest periods (stage A vs. D; stage B vs. D). Phenylpropanoid biosynthesis and pentose and glucuronate interconversions were enriched in stage A when compared to stage B. It is noteworthy that pentose and glucuronate interconversions were enriched in all two comparisons, indicating that harvest period strongly influenced pentose and glucuronate interconversions in both stage A and stage B roots. Interestingly, carotenoid biosynthesis pathway (map00906) was mainly enriched in all two comparisons based on KEGG enrichment analysis, which further suggests that different harvest period regulates secondary metabolites biosynthesis, transport, and catabolism in Astragalus (Table. S8, 9).

### Effects of harvesting period on Astragalus roots metabolic

**Fig. 8:**
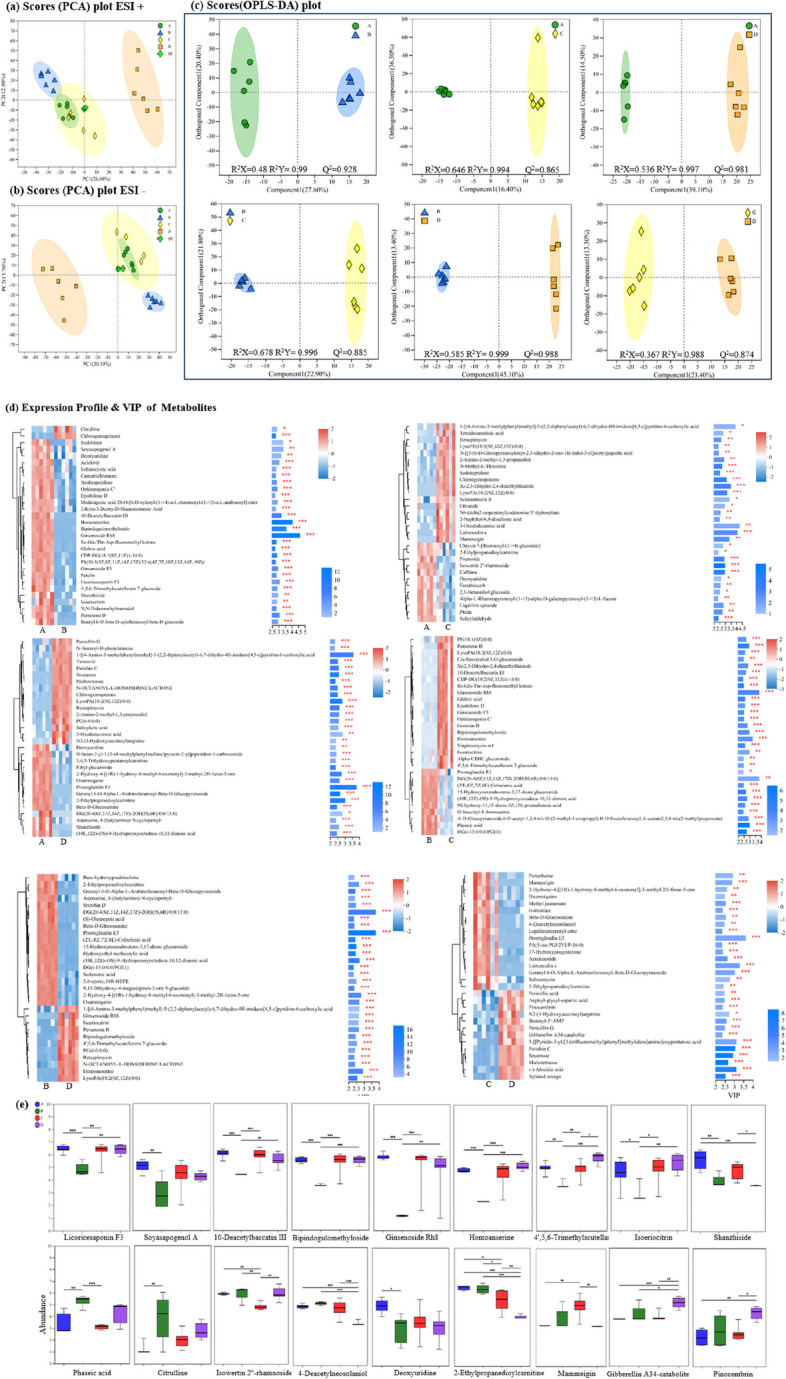
Effects of different harvest periods on Astragalus root metabolome. PCA plot in positive (**a**) and negative ion (**b**). (**c**) OPLS-DA plot of A vs. B, A vs. C, A vs. D, B vs. C, B vs. D, and C vs. D. (**d**) VIP scores analysis of A vs. B, A vs. C, A vs. D, B vs. C, B vs. D, and C vs. D. (**e**) Selected potential metabolites biomarkers. Statistical significance was determined by Student’s t-test. * *p* < 0.05, ** *p* < 0.01, *** *p* < 0.001

Metabolic variations among comparisons of period A vs. B, A vs. C, A vs. D, B vs. C, B vs. D, and C vs. D were performed using UPLC-MS/MS. A total of 1054 annotated metabolites were detected (number of ESI + and ESI- ions was 472 and 582, respectively). PCA showed significant differences among all groups in both positive and negative models (Fig. 8a, b). Clear differences were observed between stage B and D groups, and different harvest periods induced a distinction between stage A vs. D, C vs. D, and A vs. B. A total of 612 and 430 annotated metabolites were screened between stage B vs. D and A vs. B. Relative to stage A and C, 586 and 381 annotated metabolites were screened in stage D, respectively (Table. S10).

OPLS-DA and S-plot were established to validate clustering and further explore potential biomarkers of different harvest periods. First principal component explained 27.6%, 16.4%, 39.10%, 22.90%, 45.10%, and 23.40% of total variance in the comparisons of stage A vs. B, A vs. C, A vs. D, B vs. C, B vs. D, and C vs. D, and corresponding Q2 values were 0.928, 0.865, 0.981, 0.885, 0.988 and 0.874, respectively, indicating the reliability of proposed models (Fig. 8c). Ten metabolites had VIP scores > 3.0 in stage A relative to B, e.g., ginsenoside Rh8 (VIP = 4.39), homoanserine (VIP = 4.03), and bipindogulomethyloside (VIP = 3.58). Seventeen metabolites had VIP scores > 2.5 in stage A relative to D, e.g., prostaglandin E3 (VIP = 3.80) and shanzhiside (VIP = 2.59). Thirteen metabolites had VIP scores > 2.5 in stage B when compared to D (Fig. 8d), e.g., isoeriocitrin (VIP = 2.64), 4’,5,6-Trimethylscutellarein 7-glucoside (VIP = 2.54), and ginsenoside Rh8 (VIP = 2.66).

According to OPLS-DA and S-plot analysis, potential biomarkers including licoricesaponin F3, soyasapogenol A, 10-Deacetylbaccatin III, bipindogulomethyloside, ginsenoside Rh8, homoanserine, 4’,5,6-Trimethylscutellarein 7-glucoside, isoeriocitrin, and shanzhiside were significantly enhanced in stage A, C, D relative to B (*P* < 0.05). Decreased abundance of phaseic acid, citrulline, isowertin 2’’-rhamnoside, 4-Deacetylneosolaniol, deoxyuridine, and 2-Ethylpropanedioylcarnitine was observed in both stage C and D relative to B groups. The abundance of gibberellin A34-catabolite and pinocembrin enhanced significantly at stage D as the harvest period was delayed (*P* < 0.05) (Fig. 8e).

**Fig. 9:**
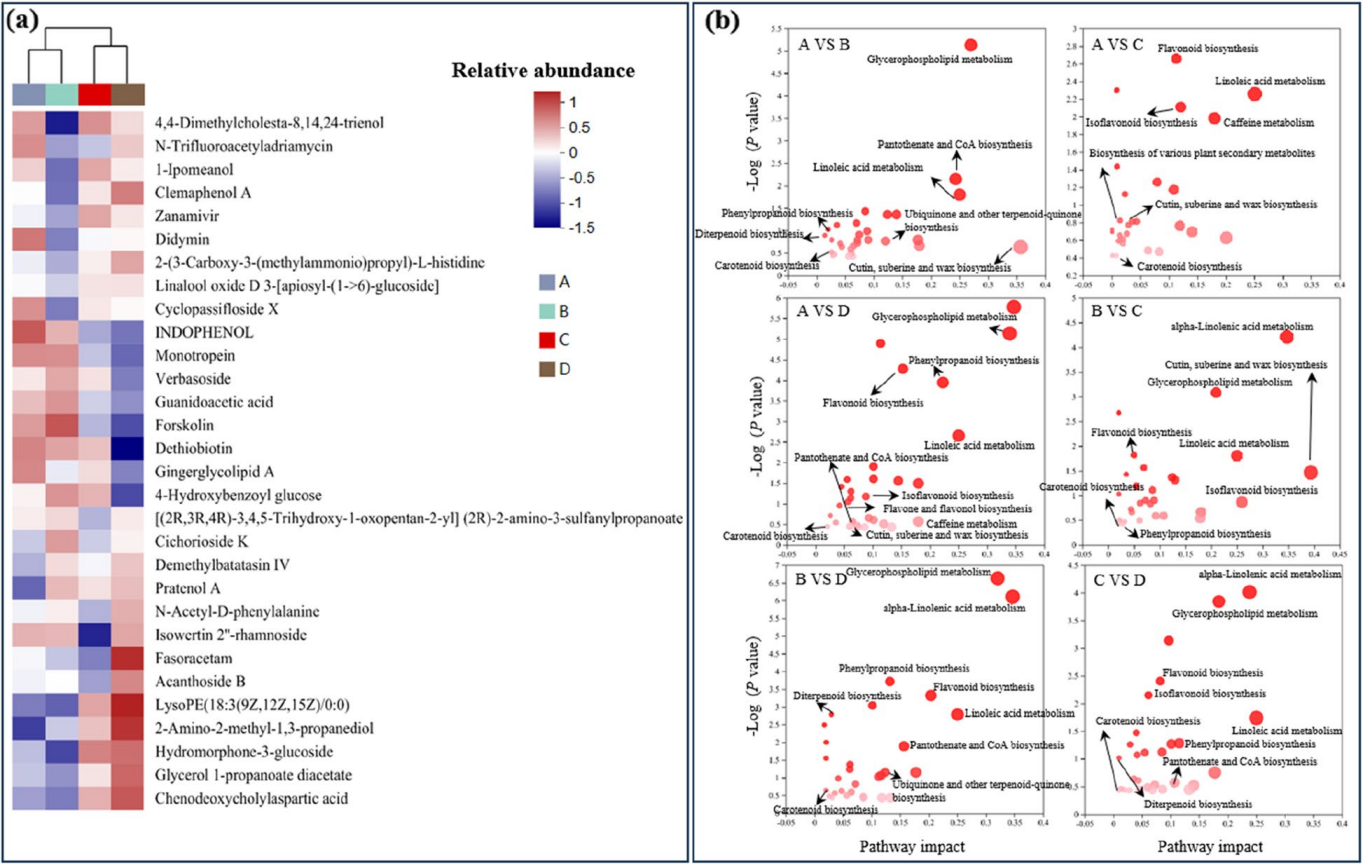
**a** Heatmap analysis for the affected metabolites (*P* < 0.05) in Astragalus roots with metabolic pathway annotations for different harvest periods A, B, C, and D (**b**). Horizontal coordinates represent the impact value on metabolic pathways. The vertical coordinate and bubble color represent the P value of enrichment analysis. Small p-value and big pathway impact factor indicate that the pathway is greatly influenced

Between different harvest periods, organic oxygen compounds, organic acids, phenylpropanoids, lipids, organic nitrogen compounds, organoheterocyclic compounds, and alkaloids were the main metabolites between the different harvest periods (Fig. 9a, Table S11). The metabolic pathways significantly impacted by different harvest periods are shown in Fig. 6b. The biological pathways involved in the metabolism of the differentially expressed metabolites and their biological roles were determined by enrichment analysis. Six perturbed metabolic pathways showed lower p-values and higher pathway impact between different harvest periods. These included pathways related to metabolism of glycerophospholipid metabolism (map00564), linoleic acid metabolism (map00591), caffeine metabolism (map00232), alpha-linolenic acid metabolism (map00592), flavonoid biosynthesis (map00941), and phenylpropanoid biosynthesis (map00940). Isoflavonoid biosynthesis (map00943) were relatively affected by different harvest periods, and Cutin, suberine and wax biosynthesis (map00073) were significantly enhanced in stages A vs. B and B vs. C (Fig. 9b).

### Integrated analysis of transcriptome and metabolome

**Fig. 10:**
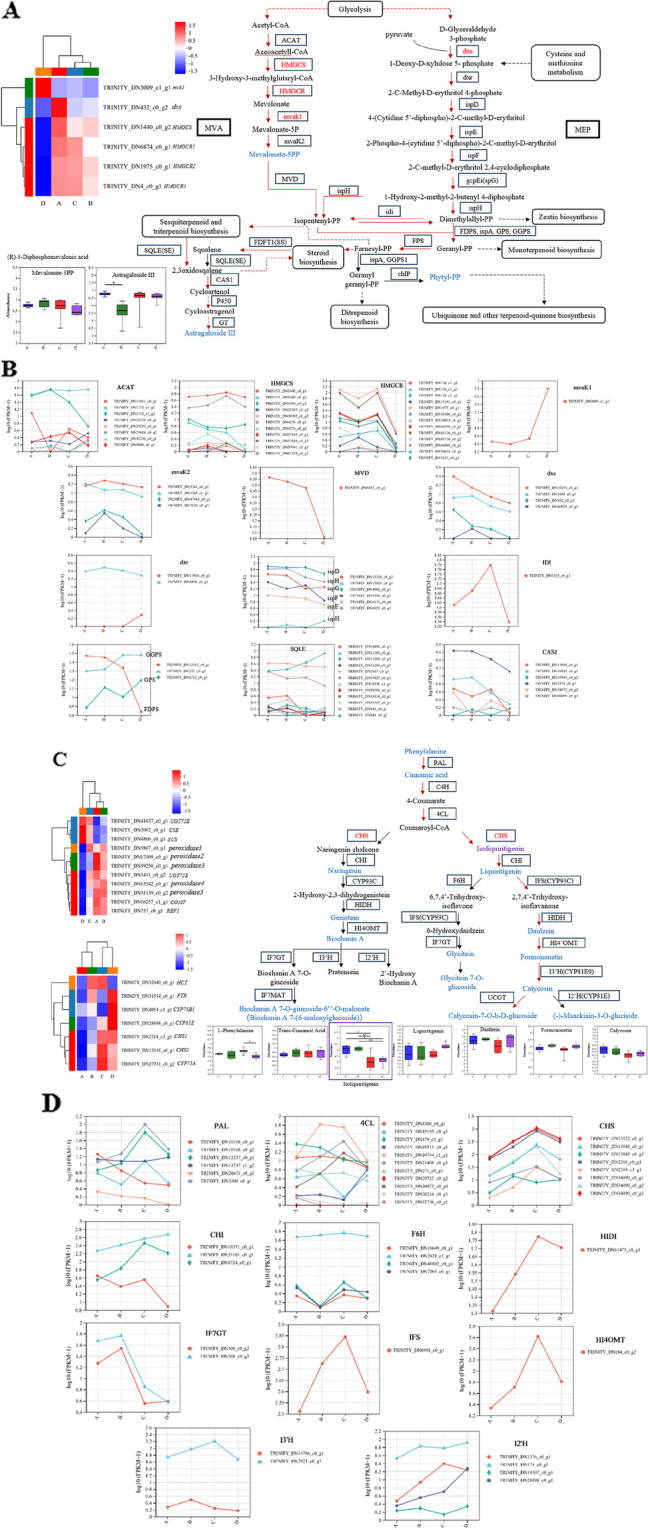
Affected metabolic pathways indicated by RNA extracted from Astragalus roots under the different harvest periods. Significant DEGs involved in terpenoid backbone biosynthesis, phenylpropanoid biosynthesis, and flavonoid biosynthesis & Isoflavonoid biosynthesis under different harvest periods in Astragalus roots. The enzymes confirmed by transcriptomics data are shown in black. Red indicates enzymes with significant differential expression at different harvest periods. Blue letters indicate the significant differential metabolites in Astragalus roots from different harvest periods. The pathway adapted from KEGG. The heatmap was generated from the log2-fold change (log10FC) mean value calculated from three replicates of RNA-Seq data. **A**. The Terpenoids biosynthesis pathway. **B**, **D**. The expression of thegenes (enzymes) in the pathway measured by FPKM value. **C**.The phenylpropanoid, flavonoids, and isoflavonoids biosynthesis pathway

Many DEGs that showed distinct expression patterns between different harvest periods were related to lipid metabolism, amino acid metabolism, and biosynthesis of other secondary metabolites. Interestingly, the 6, 11, and 7 DEGs annotated to terpenoid backbone biosynthesis, phenylpropanoid biosynthesis, flavonoid biosynthesis, and Isoflavonoid biosynthesis were significantly upregulated or downregulated in harvest periods A, B C, and D, respectively. In particular, 24 genes in all metabolic pathways were significantly altered during stage D, which further suggests that harvest period regulates a variety of metabolism in Astragalus (Fig. 10A, C).

**Fig. 11:**
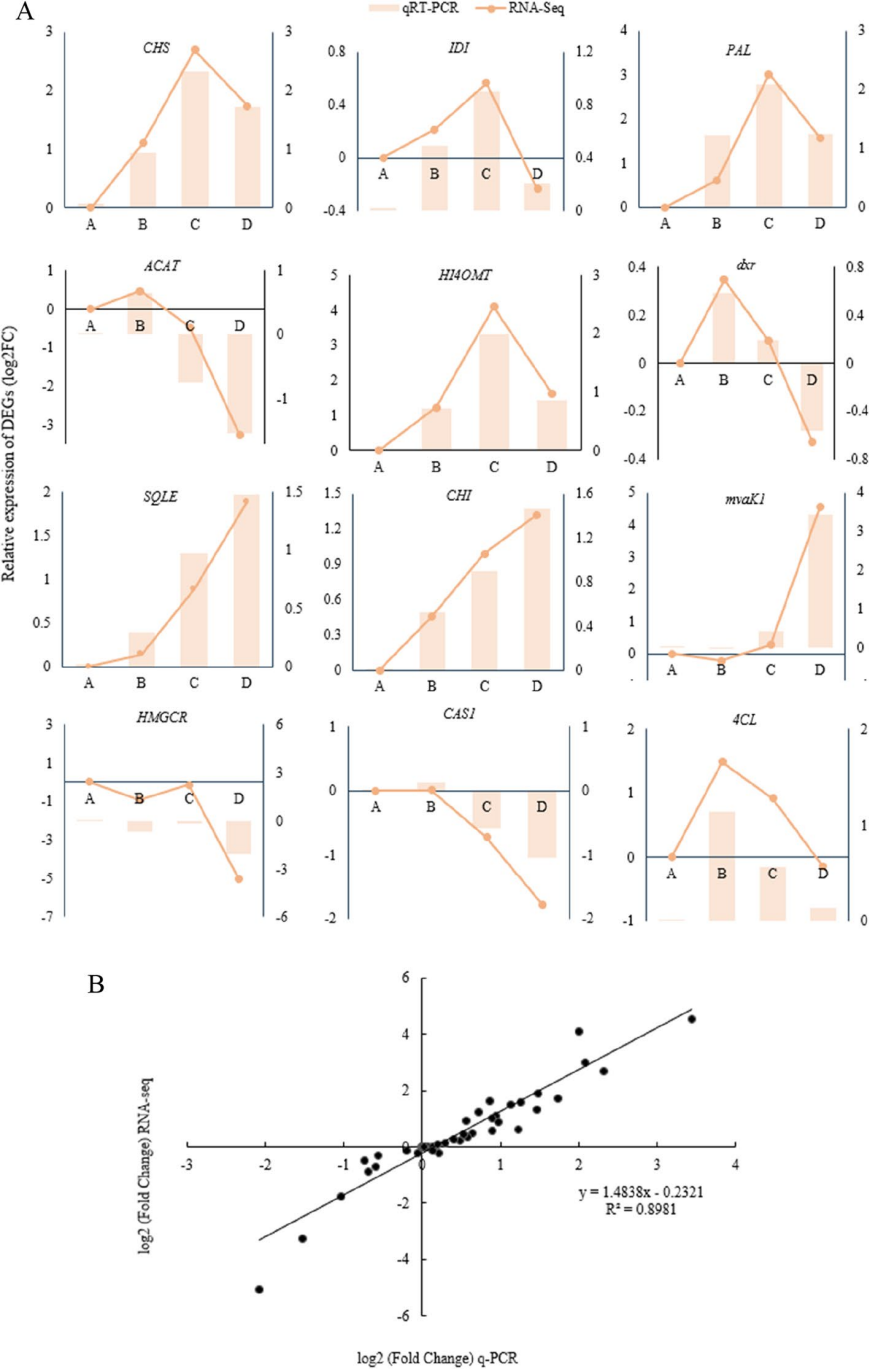
**A** The integrated analysis of qRT–PCR and RNA-Seq validation for 12 genes related to the synthesis of astragaloside IV and calycosin 7-O-β-D-glucopyranoside in Astragalus. **B** Linear regression between qRT–PCR and RNA-Seq.

The terpenoid backbone biosynthesis pathway was the base of the biosynthesis of triterpenoid saponins [33]. In the terpenoid backbone biosynthesis pathway of lipid metabolism (Fig. 11b), only two mevalonate kinase (*mvaK1*), geranyl diphosphate synthase (*GPS*) were upregulated 19.36 and 1.26-fold in stage D. The other seventeen structural DEGs, including 4 acetyl-CoA C-acetyltransferase (*AACT*), 10 hydroxymethylglutaryl-CoA synthase (*HMGCS*), 13 hydroxymethylglutaryl-CoA reductase (*HMGCR*), 4 phosphomevalonate kinase (*mvaK2*), 1 diphosphomevalonate decarboxylase (*MVD*), 4 1-deoxy-D-xylulose-5-phosphate synthase (*dxs*), 1 1-deoxy-D-xylulose-5-phosphate reductoisomerase (*dxr*), 1 2-C-methyl-D-erythritol 4-phosphate cytidylyltransferase (*ispD*), 1 4-hydroxy-3-methylbut-2-en-1-yl diphosphate reductase (*ispH*), 1 (E)-4-hydroxy-3-methylbut-2-enyl-diphosphate synthase (*ispG*), 1 2-C-methyl-D-erythritol 2,4-cyclodiphosphate synthase (ispF), 1 4-diphosphocytidyl-2-C-methyl-D-erythritol kinase (*ispE*), 1 isopentenyl-diphosphate Delta-isomerase (*IDI*), 1 farnesyl diphosphate synthase (*FDPS*), 8 squalene monooxygenase (*SQLE*), and 5 cycloartenol synthase (*CAS1*), were generally downregulated in stage D. We found some high levels of astragalosides in stage C, where MVA pathway was dominant. We discovered six isoforms for CAS1, DN2374, DN15048, DN16943c0-g1, DN16943c0-g2, DN58972 and DN60699. Among these, DN16943c0-g2 and DN60699 were expressed specifically in different harvest periods. DN15048 and DN58972 were highly expressed in stage C, whereas DN2374 and DN16943c0-g1 were similarly expressed in four periods. On the other hand, the content of ~ 8 monoterpenoids and diterpenoids showed significant changes in leaf than in different harvest periods, especially in stage D (VIP > 1 and fold change ≥ 2). This suggested that monoterpenoids and diterpenoids are synthesized through the MEP/DOXP pathway, which is dominant in harvest period D (Fig. S2). Overall, fatty acid synthesis in Astragalus plant roots was inhibited under harvest period D, since the transcriptional abundance of most structural DEGs involved in this pathway was significantly reduced.

To further explore the effect of different harvest periods on structural genes of Isoflavonoid biosynthesis, the transcriptional abundance of 11 DEGs involved in Isoflavonoid biosynthesis was visualized (Fig. 10D). The 11 DEGs encode 6 phenylalanine ammonia-lyase (*PAL*), 11 4-coumarate–CoA ligase (*4CL*), 8 chalcone synthase (*CHS*), 3 chalcone isomerase (*CHI*), 4 flavonoid 6-hydroxylase (*F6H*), 1 2-hydroxyisoflavanone dehydratase (HIDI), 2 isoflavone 7-O-glucosyltransferase (*IF7GT*), 1 2-hydroxyisoflavanone synthase (*IFS*), 1 2,7,4’-trihydroxyisoflavanone 4’-O-methyltransferase / isoflavone 4’-O-methyltransferase (HI4OMT), 2 isoflavone 3’-hydroxylase (*I3’H*), and 4 isoflavone/4’-methoxyisoflavone 2’-hydroxylase (I2’H) genes. Notably, almost all of the structural DEGs were significantly downregulated in harvest period D to varying degrees, especially *CHS*, *IF7GT*, *IFS*, and *HI4OMT* coding genes, which were downregulated more than 3-fold, indicating that delays in harvesting comprehensively affected Isoflavonoid metabolism in Astragalus plants. We identified isoliquiritigenin that was not reported in Astragalus before. It confirmed the activity of *CHI* in liquiritigenin synthesis process. Isoliquiritigenin, the critical metabolite in the pathway, was first identified f*or* Astragalus by *o*ur current study. The relative contents of isoliquiritigenin in this branch were mostly higher in harvest periods A and B than in other two periods (Fig. 10C).

### Integrated analysis of 12 secondary metabolism-related genes with RNA-Seq and qRT-PCR

The 12 genes related to the synthesis of astragaloside IV and calycosin 7-O-β-D-glucopyranoside were verified by qRT-PCR and RNA-Seq. The R^2^ of the linear regression between gene relative expression and transcriptome data was 0.8981, which proved the reliability of transcriptome data (Fig. 11).

### Correlation analysis of medicinal materials phenotype and quality with genes and metabolites related to phenylpropanoid, flavonoid, and isoflavonoid biosynthesis

**Fig. 12:**
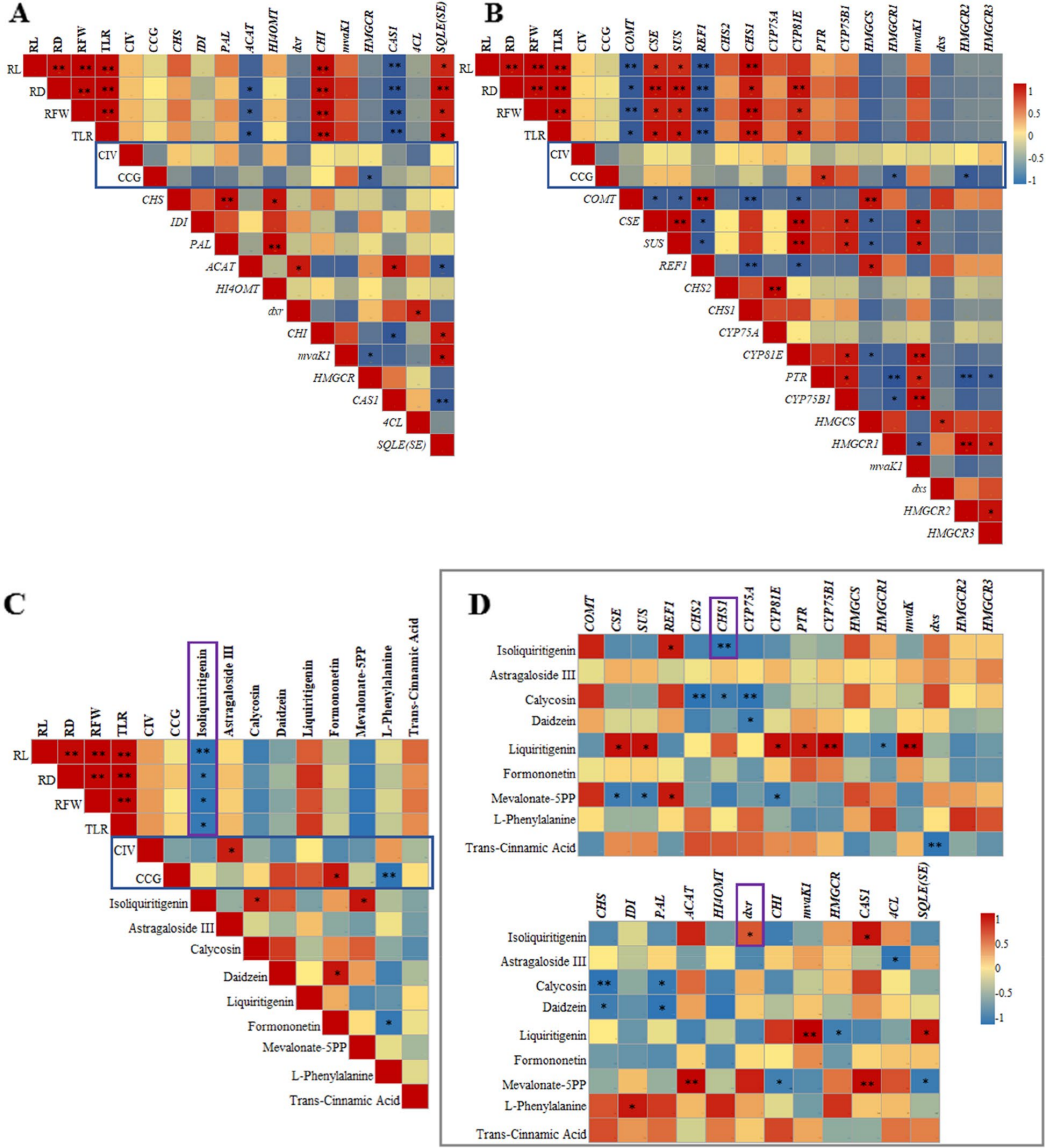
Correlation analysis of root phenotype and content of two main active components with genes and metabolites related to phenylpropanoid biosynthesis, flavonoid biosynthesis, and isoflavonoid biosynthesis in different harvest periods. **A** Pearson correlation analysis of 12 genes related to the synthesis of two main active components by qRT-PCR. **B** Pearson correlation analysis of 16 degs with the root phenotype and the content of the two main active components. **C** Pearson correlation analysis of 9 metabolites with the root phenotype and the content of the two main active components. **D** Pearson correlation analysis of degs and 9 metabolites by qRT–PCR and UHPLC-Exactive HF-X. Abbreviations Capital letters represent respectively: RL- Root length, RD- Root diameter, RFW- Root fresh weight, TLR- Thick lateral root, CIV- Content of astragaloside IV, CCG- Content of calycosin 7-O-β-D-glucopyranoside. *P < 0.05, **P < 0.01

 Pearson correlations of the expression levels of 12 astragaloside IV and calycosin 7-O-β-D-glucopyranoside synthesis-related genes and 16 degs from qRT-PCR showed that *HMGCR*, *HMGCR1*, and *HMGCR2* had a significant negative correlation with the content of calycosin 7-O-β-D-glucopyranoside (*P* < 0.05), *PTR* had a significant positive correlation with the content of calycosin 7-O-β-D-glucopyranoside (*P* < 0.05). *ACAT*, *CHI*, *CAS1*, *SE*, *COMT*, *CSE*, *SUS*, *REF1*, *CHS1*, *CYP81E* 13 genes had a strong correlation with the root phenotypic traits root length, root diameter, root fresh weight, and thick lateral root of Astragalus (*P* < 0.05, *P* < 0.01) (Figs. 9B and 12A, Table S12, S13). In addition, *CHS* and *PAL* had a positive correlation with *HI4OMT* (*P* < 0.01, *P* < 0.05) (Fig. 12A, Table S12). Furthermore, *CAS1* were strongly correlated with *SE* (*P* < 0.01) (Fig. 12A, Table S12).

Metabolite isoliquiritigenin had a strong correlation with the root phenotypic traits root length, root diameter, root fresh weight, and thick lateral root of Astragalus (*P* < 0.05, *P* < 0.01). Astragaloside III had a significant positive correlation with the content of astragaloside IV (*P* < 0.05), formononetin and L-Phenylalanine metabolites had a strong correlation with the the content of calycosin 7-O-β-D-glucopyranoside (*P* < 0.01, *P* < 0.05) (Fig. 12C, Table S14). Moreover, the Pearson correlation between 9 metabolites and 28 genes across all samples showed that *CSE*, *SUS*, *CYP81E*, *PTR*, *CYP75B1*, *mvaK*, *mvaK1*, and *SE* had a significant positive correlation with liquiritigenin (*P* < 0.05, *P* < 0.01), *HMGCR* and *HMGCR1* had a significant negative correlation with the content of liquiritigenin (*P* < 0.05) (Fig. 12D, Table S15, S16). *IDI* had a significant positive correlation with L-phenylalanine (*P* < 0.05), and *4CL* had a significant negative correlation with Astragaloside III (*P* < 0.05) (Fig. 12D). Hence, *CHS*, *CHI*, *IDI*, *PAL* and *4CL* genes played an important role in the accumulation of phenylpropanoids, flavonoids and isoflavones pathway.

Transcriptomic and metabolomic analyses showed that the phenylpropanoid biosynthesis, flavonoid biosynthesis, and isoflavonoid biosynthesis pathway play an important role in the difference between different harvest periods. Based on these results, the yield of Astragalus and the accumulation mechanisms of astragaloside IV and calycosin 7-O-β-D-glucopyranoside in stage D were primarily due to the overexpression of key genes *ACAT*, *COMT*, *SUS*, *CHS*, *CHI*, *IDI*, *CYP81E*, *PAL* and *4CL* in the three biosynthetic pathways. L-phenylalanine was decreased, and liquiritigenin was produced, which led to the formation of the flavonoid backbone. First, the low expression of *4CL* in the phenylpropane pathway led to a decrease in L-phenylalanine. Subsequently, the low expression of rate-limiting enzymes, such as *CHI* and *CHS* further promoted the accumulation of liquiritigenin in the midstream pathway. The downstream pathway focused on modifying the flavonoid backbone. Finally, astragaloside III, calycosin, formononetin, daidzein, CIV, and CCG showed significant accumulation because of the overexpression of *ACAT*, *COMT*, *CHS*, *SUS*, *IDI*, and *4CL* genes (Figs. 10 and 12). The dxr and CHS1 genes upstream of the terpenoids biosynthesis pathway and the phenylpropanoid, flavonoids, and isoflavonoids biosynthesis pathway significantly regulate the synthesis of the metabolite isoliquiretigin at different periods. CAS1 and REF1 genes also play a significant role in the synthesis of isoliquiretigin. (Fig. 12D).

## Discussion

The molecular mechanisms underlying Astragalus root development are still poorly understood. The initiation of harvest period is the critical stage that determines crop yield and active ingredient content. It is difficult to elucidate the regulatory mechanism that determines the initiation or the tipping point of the conversion from early harvest stage to the late harvest stage and its core regulators [34]. Conventional transcriptomic analyses have provided insights into temporal gene expression associated with Astragalus root development [35–37], however, the approach is limited by a number of steady-state gene expression profiles. Compared with previous transcriptomic studies, the combined analysis of phenotype, transcriptome and metabolome in our study has yielded considerably more comprehensive genes and metabolites associated with the root harvest period developmental stages. In this study, we investigated the effect of different harvesting periods on the accumulation pattern of important metabolites in the roots of the medicinal and edible plant Astragalus. Significant differences in phenotypic traits and relative abundance of metabolites in roots at different harvesting periods with delayed harvesting. These findings are consistent with the Heracles Neo by Yu, and Wang et al. [38, 39]. The abundance of phenylalanine metabolites upstream of the phenylpropanoid, flavonoids, and isoflavonoids biosynthesis pathway varied as C > A > B > D, whereas the calycosin 7-O- β -D-glucopyranoside content synthesized by this pathway changed D > B > A > C. This indicating that the upregulation of phenylpropanoid played an inhibitory role in the synthesis of calycosin 7-O-β-D-glucopyranoside, with the highest content of calycosin 7-O- β -D-glucopyranoside during period D. Downstream of Terpenoids biosynthesis pathway, Astragaloside III metabolite abundance changed A > C > D > B, while the content of Astragaloside IV synthesized by this pathway changed C > A > D > B. The trend of the two metabolites were roughly the same, and the content of Astragaloside IV was the highest in the period C. Considering the higher yield of medicinal materials in period D, it was determined that period D was better for harvesting. It is worth noting that decreased *HMGCS*, *HMGCR*, *dxs*, *IDI*, *CAS1*, *CHS*, *IFS*, and *IOMT* levels in root were observed with period D.

Root transcriptome was performed to further understand the effect of harvesting periods on root-associated metabolic processes. The period of harvesting has a significant effect on isofavones accumulation, with delayed harvesting promoting the accumulation of calycosin 7-O-β-D-glucopyranoside in roots. A combined transcriptome metabolome analysis confirmed that the isoflavones content of six-year-old Astragalus roots was significantly higher than that of two-year-old [5]. Similarly, significant effect on the root transcriptome was observed with delayed harvesting in this study. However, excessive delay in harvesting (e.g., period D) in the same year had a promoted effect on isoflavone accumulation. Most importantly, we have identified the critical period (period D) affecting yield and active ingredient content accumulation and confirmed that genes such as *ACAT*, *COMT*, *CHS*, *SUS*, *IDI*, and *4CL*, and metabolites such as isoglycyrrhizin, astragaloside III, l-phenylalanine, glycyrrhizin, and glycyrrhizin are the core regulatory genes and metabolites for herb yield and content at the harvesting period, which is of great significance for the rational harvesting of Astragalus medicinal materials.

The expression data throughout the four stages exhibited high reproducibility and differential expression during the growth process in Astragalus harvest period. On the basis of PCA analysis, the root growth stages were clustered into three prominent groups, signifying the differences in the gene expression profiles from one stage to another (Fig. 3). These clusters depicted similar kinds of gene expression during the progression from the early harvest to the late harvest roots. The results of GO analysis emphasized the significant functions of several biological processes during root developmental progression. Furthermore, the biological functions of these differently expressed genes in different harvest periods were drawn by GO analysis, which highlighted the significant changes in various metabolic processes, e.g. carbohydrate metabolic processes, cell wall metabolism, transcription regulator activity, protein metabolic processes, secondary metabolic processes and response to stress, and these findings are in line with previous findings [40–42]. The data obtained during the root harvesting process validated and established the chronology of gene expression events and revealed that the onset of downregulation-enriched biological functions preceded that of activation. The first state conversion began from period B to period C and was represented by the transcriptional regulators, consistent with the apparent changes in astragaloside IV and calycosin 7-O-β-D-glucopyranoside contents during this period. In order to determine potential biomarkers of the harvest period of medicinal materials and better investigate the molecular mechanisms of regulating the growth of Astragalus root, we performed the untargeted metabolome method and identified period C as the tipping point of root significant changes in differential metabolites (Fig. 10), which is also consistent with the progression of root development and the dynamics of gene expression (Figs. 2B and 10C and D).

Furthermore, isoliquiritigenin was found to be one of the important metabolites in the secondary metabolic pathway of Astragalus and might have an important influence on the content of active ingredients in medicinal materials during harvest. Down-regulation of isoliquiritigenin significantly inhibited the increase of root biomass (Figs. 2 and 10). Previously, lquiritigenin is often used as a precursor in subsequent reactions to form flavonoids, such as flavones, dihydrofavones, and isoflavones [43]. Therefore, isoliquiritigenin could be the limited factor for initiating lquiritigenin. Physico-chemical characteristics of isoliquiritigenin and related potential metabolic markers should be taken into consideration in further investigation. By transcriptome metabolome association analysis, we found that several known root development related genes and metabolites can be regulated by isoliquiritigenin (Fig. 10). These results confirmed that *REF*, *CHS1*, *dxr*, and *CAS1* played an important role in root development during harvest by regulating multiple metabolic and signaling pathways (Fig. 12D).

To further investigate the mechanism of root development during the harvest of Astragalus, we performed a phenotypic, transcriptome, and metabolomics association analysis, and identified some key genes and metabolites related to the development stage from the early harvest to the late harvest (Fig. 12). These genes and metabolites can play synergistic roles in regulating harvest each period, and several members of these genes were found to be involved in different aspects of root development in different plants [44–47]. Our results indicated that the phenotypic and transcriptome metabolome combined correlation analysis, except for the establishment of co-expression modules, can be extremely useful to investigate the molecular mechanisms of root development. However, to illustrate the details of these genes, it is necessary to conduct further functional investigations for each component in the relevance analysis.

This study demonstrated that delayed harvest effectively increases the yield and active ingredient content of Astragalus. Integrated root transcriptome and metabolome indicated that the delay of harvest time mainly altered transcription levels of genes involved in terpenoid backbone biosynthesis, thiamine metabolism, phenylpropanoid biosynthesis, flavonoid biosynthesis, and isoflavonoid biosynthesis, and modulated the abundance of metabolic biomarkers of licoricesaponin F3, isoeriocitrin, (R)-5-Diphosphomevalonic acid, astragaloside III, l-Phenylalanine and formononetin. Further, RT-PCR validated that the delay of harvest time significantly affected the key genes involved in secondary metabolism in each period, and promoted the growth of root biomass and the synthesis of two main secondary metabolites. Insight into how the harvest period affects the yield of medicinal roots and promotes the synthesis of active ingredients would contribute to improving the yield of Astragalus medicinal materials and the content of medicinal ingredients in actual production and lay the foundation for subsequent molecular breeding research. In summary, our work gives new insights into the thedynamics and architecture of the root development regulatory network and provides a valuable data set for mining other genes associated with root growth and development in root crops like Astragalus during harvest period. Our work is another successful example of determining the optimal harvest time of medicinal materials using different harvest times. In particular, isoliquiritigenin at different harvest stages are the key metabolites that affect the content of active ingredients in Astragalus.

## Conclusions

Combined phenotypic, transcriptomic, and metabolomics analysis results have greatly clarified the changing trends of the metabolite profile and transcriptome profile at different harvest periods. Maker metabolites, including astragaloside III, isoliquiritigenin, liquiritigenin, formononetin, daidzein, astragaloside IV, and calycosin 7-O-β-D-glucopyranoside, were highly enriched in period D. Our analyses further indicate that the regulation of key genes (*HMGCS*, *HMGCR*, *mvaK*, *dxs*, *4CL*, *CHS*, *CHI*, and *SQLE*) was the main reason for the high accumulation of isoflavones and triterpenoid saponins substances in period D.

### Supplementary Information

**Additional file 1: Figure S1. **PCA plot of roots transcriptome profiles.

**Additional file 2: Figure S2. **VIP scores analysis of monoterpenoids and diterpenoids.

**Additional file 3: Table S1.** The quality control of RNA sequencing of liver samples.

**Additional file 4: Table S2.** DEGs and annotated information by period.

**Additional file 5: Table S3. **STEM profile information.

**Additional file 6: Table S4. **KEGG annotation information for CLUSTER0 and CLUSTER1.

**Additional file 7: Table S5. **DEGs expression and annotation information on ipath.

**Additional file 8: Table S6. **Annotated information on GO enrichment analysis of DEGs in periods A and D.

**Additional file 9: Table.S7. **Annotated information on GO enrichment analysis of DEGs in periods B and D.

**Additional file 10: Table S8. **Annotated information on KEGG enrichment analysis of DEGs in periods A and D.

**Additional file 11: Table S9. **Annotated information on KEGG enrichment analysis of DEGs in periods B and D.

**Additional file 12: Table S10. **Differential metabolite statistics.

**Additional file 13: Table S11. **TOP30 metabolite information and expression abundance at different time periods.

**Additional file 14: Table S12.** Pearson correlation analysis of 12 significantly differentially expressed genes with the root phenotype and the content of the two main active components by qRT–PCR.

**Additional file 15: Table S13.** Pearson correlation analysis of 16 significantly differentially expressed genes with the root phenotype and the content of the two main active components.

**Additional file 16: Table S14.** Pearson correlation analysis of 9 metabolites with the root phenotype and the content of the two main active components.

**Additional file 17: Table S15.** Pearson correlation analysis of 16 Degs and 9 metabolites by FPKM and UHPLC-Exactive HF-X.

**Additional file 18: Table S16.** Pearson correlation analysis of 12 genes and 9 metabolites by FPKM and UHPLC-Exactive HF-X

**Additional file 19: Table S17.** qRT–PCR primers used in this study.

**Additional file 20: Supplementary data1. **Functional annotations and TPM values of all transcripts.

**Additional file 21: Supplementary data2. **De novo assembled unigenes in FASTA format.

## Data Availability

Sequence data that support the findings of this study have been deposited in the European Nucleotide Archive with the primary accession code PRJNA1068253. Data is provided within the manuscript or supplementary information files.

## Abbreviations

DEGs: Differentially expressed genes
VIP: Variable Importance in the Projection
KEGG: Kyoto encyclopedia of genes and genomes
GO: Gene ontology
FPKM: Fragments Per Kilobase per Million
FC: Fold change
qRT-PCR: Real-time quantitative PCR
OPLS-DA: Orthogonal Partial Least Squares Discriminant Analysis
