# Supplementary material for: Integrated phenotypic, transcriptomics and metabolomics: growth status and metabolite accumulation pattern of medicinal materials at different harvest periods of Astragalus Membranaceus Mongholicus
Source: BMC Plant Biol. 2024 May 3;24:358. doi: 10.1186/s12870-024-05030-7 (PMC11067282; doi:10.1186/s12870-024-05030-7)
Supplement: Supplementary file 1 — Additional file 1: Figure S1. PCA plot of roots transcriptome profiles. [file 12870_2024_5030_MOESM1_ESM.docx]

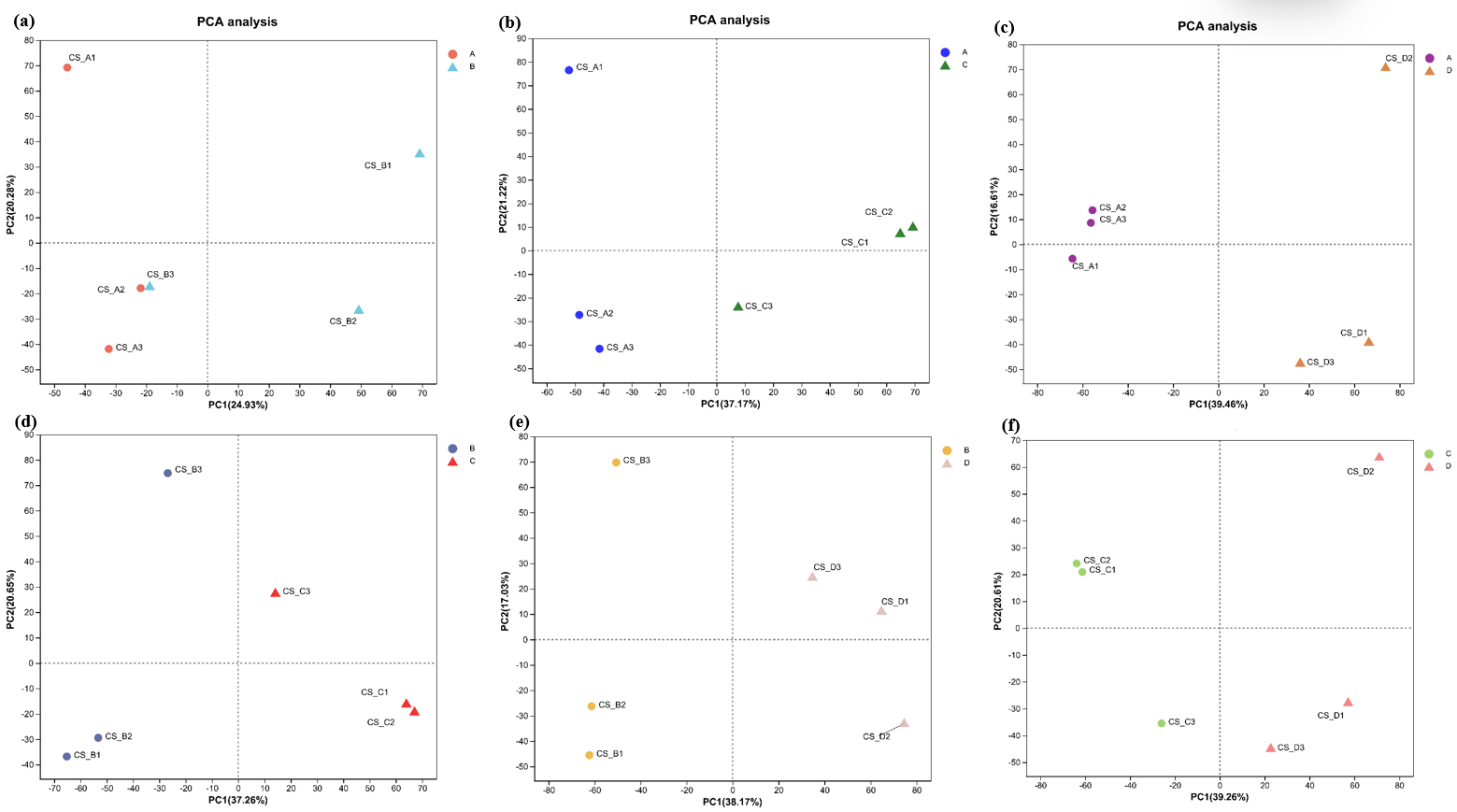


**Figure S1.** PCA plot of roots transcriptome profiles. (a) Stage A vs. B. (b) Stage A vs. C. (c) Stage A vs. D. (d) Stage B vs. C. (e) Stage B vs. D. (f) Stage C vs. D.
