# Supplementary material for: Integrated phenotypic, transcriptomics and metabolomics: growth status and metabolite accumulation pattern of medicinal materials at different harvest periods of Astragalus Membranaceus Mongholicus
Source: BMC Plant Biol. 2024 May 3;24:358. doi: 10.1186/s12870-024-05030-7 (PMC11067282; doi:10.1186/s12870-024-05030-7)
Supplement: Supplementary file 2 — Additional file 2: Figure S2. VIP scores analysis of monoterpenoids and diterpenoids. [file 12870_2024_5030_MOESM2_ESM.docx]

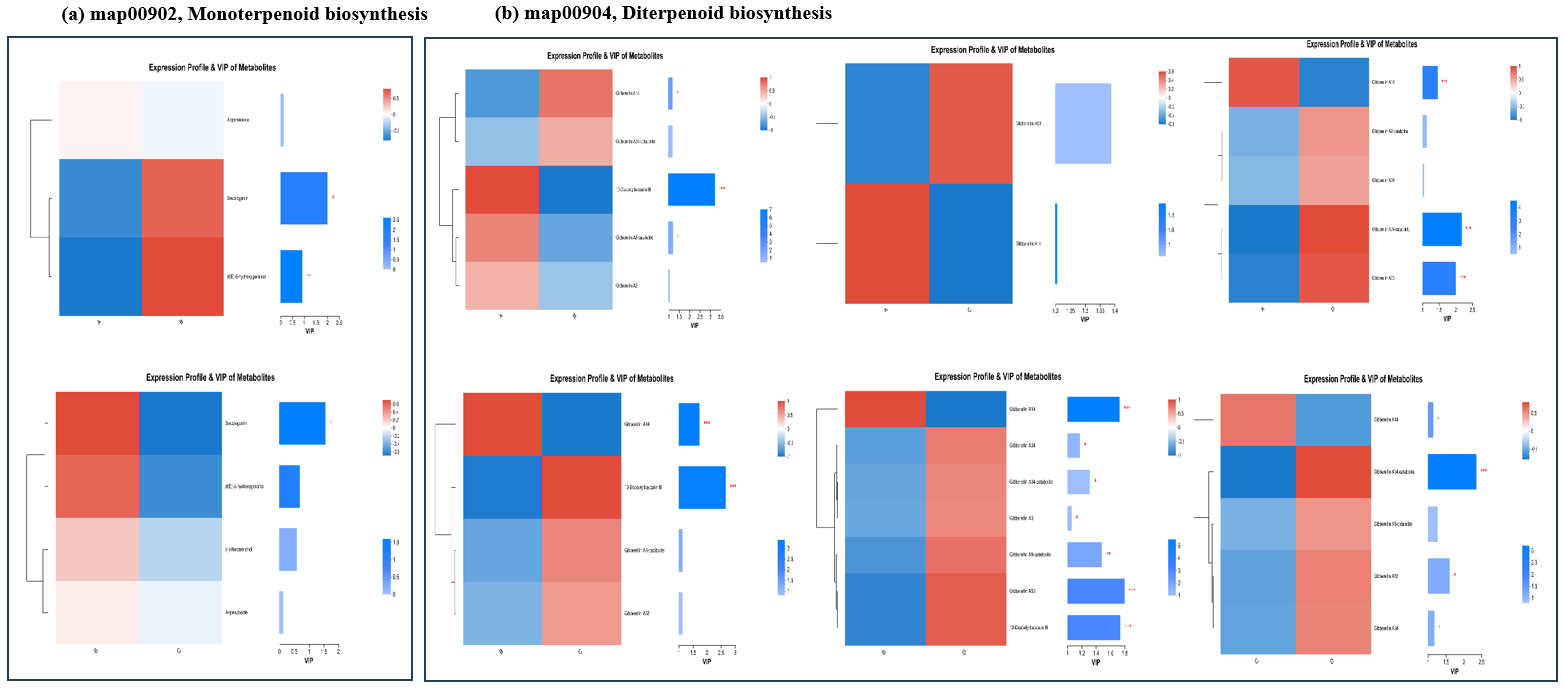


**Figure S2.** VIP scores analysis of monoterpenoids and diterpenoids. Statistical significance was determined by Student's t-test. * p < 0.05, ** p < 0.01.
