# Supplementary material for: Integrated phenotypic, transcriptomics and metabolomics: growth status and metabolite accumulation pattern of medicinal materials at different harvest periods of Astragalus Membranaceus Mongholicus
Source: BMC Plant Biol. 2024 May 3;24:358. doi: 10.1186/s12870-024-05030-7 (PMC11067282; doi:10.1186/s12870-024-05030-7)
Supplement: Supplementary file 3 — Additional file 3: Table S1. The quality control of RNA sequencing of liver samples. [file 12870_2024_5030_MOESM3_ESM.docx]

Table S1 The quality control of RNA sequencing of liver samples

| Group | Sample | Clean reads | Error rate(%) | Q30(%) | GC content(%) |
| --- | --- | --- | --- | --- | --- |
| A | CS_A1 | 42781556 | 0.026 | 93.23 | 43.81 |
|  | CS_A2 | 58417274 | 0.0263 | 92.96 | 43.81 |
|  | CS_A3 | 42863720 | 0.0258 | 93.46 | 45.17 |
| B | CS_B1 | 48341560 | 0.0261 | 93.13 | 43.17 |
|  | CS_B2 | 44288732 | 0.0254 | 93.82 | 43.07 |
|  | CS_B3 | 46043178 | 0.0262 | 92.94 | 43.7 |
| C | CS_C1 | 46892762 | 0.0266 | 92.67 | 43.53 |
|  | CS_C2 | 44072564 | 0.026 | 93.18 | 43.48 |
|  | CS_C3 | 45562006 | 0.0259 | 93.28 | 44.13 |
| D | CS_D1 | 42636374 | 0.0261 | 93.04 | 43.34 |
|  | CS_D2 | 54852934 | 0.0264 | 92.83 | 43.41 |
|  | CS_D3 | 44794028 | 0.0255 | 93.76 | 43.71 |
