# Supplementary material for: Integrated phenotypic, transcriptomics and metabolomics: growth status and metabolite accumulation pattern of medicinal materials at different harvest periods of Astragalus Membranaceus Mongholicus
Source: BMC Plant Biol. 2024 May 3;24:358. doi: 10.1186/s12870-024-05030-7 (PMC11067282; doi:10.1186/s12870-024-05030-7)
Supplement: Supplementary file 14 — Additional file 14: Table S12. Pearson correlation analysis of 12 significantly differentially expressed genes with the root phenotype and the content of the two main active components by qRT–PCR. [file 12870_2024_5030_MOESM14_ESM.docx]

Table S12. Pearson correlation analysis of 12 significantly differentially expressed genes with the root phenotype and the content of the two main active components by qRT–PCR

|  | RL | RD | RFW | TLR | CIV | CCG | CHS | IDI | PAL | ACAT | HI4OMT | dxr | CHI | mvaK1 | HMGCR | CAS1 | 4CL | SQLE(SE) |
| --- | --- | --- | --- | --- | --- | --- | --- | --- | --- | --- | --- | --- | --- | --- | --- | --- | --- | --- |
| RL | 1 | 0.97** | 1.00** | 0.98** | 0.46 | 0.13 | 0.73 | 0.04 | 0.64 | -0.87 | 0.51 | -0.63 | 0.98** | 0.71 | -0.56 | -0.97** | -0.32 | 0.89* |
| RD | 0.97** | 1 | 0.98** | 1.00** | 0.44 | 0.32 | 0.53 | -0.21 | 0.44 | -0.95* | 0.28 | -0.77 | 0.97** | 0.86 | -0.69 | -1.00** | -0.47 | 0.97** |
| RFW | 1.00** | 0.98** | 1 | 0.99** | 0.47 | 0.17 | 0.67 | -0.03 | 0.59 | -0.90* | 0.45 | -0.68 | 0.98** | 0.76 | -0.59 | -0.99** | -0.37 | 0.92* |
| TLR | 0.98** | 1.00** | 0.99** | 1 | 0.48 | 0.26 | 0.57 | -0.16 | 0.49 | -0.95* | 0.34 | -0.76 | 0.97** | 0.83 | -0.65 | -1.00** | -0.47 | 0.96* |
| CIV | 0.46 | 0.44 | 0.47 | 0.48 | 1 | -0.53 | 0.42 | 0.1 | 0.52 | -0.63 | 0.47 | -0.68 | 0.29 | 0.19 | 0.27 | -0.47 | -0.76 | 0.32 |
| CCG | 0.13 | 0.32 | 0.17 | 0.26 | -0.53 | 1 | -0.47 | -0.79 | -0.61 | -0.25 | -0.71 | -0.25 | 0.32 | 0.7 | -0.89* | -0.27 | -0.06 | 0.51 |
| CHS | 0.73 | 0.53 | 0.67 | 0.57 | 0.42 | -0.47 | 1 | 0.72 | 0.98** | -0.37 | 0.95* | -0.07 | 0.63 | 0.04 | 0.04 | -0.55 | 0.15 | 0.33 |
| IDI | 0.04 | -0.21 | -0.03 | -0.16 | 0.1 | -0.79 | 0.72 | 1 | 0.77 | 0.36 | 0.86 | 0.57 | -0.08 | -0.67 | 0.6 | 0.19 | 0.57 | -0.42 |
| PAL | 0.64 | 0.44 | 0.59 | 0.49 | 0.52 | -0.61 | 0.98** | 0.77 | 1 | -0.31 | 0.99** | -0.05 | 0.51 | -0.08 | 0.2 | -0.47 | 0.1 | 0.22 |
| ACAT | -0.87 | -0.95* | -0.90* | -0.95* | -0.63 | -0.25 | -0.37 | 0.36 | -0.31 | 1 | -0.16 | 0.93* | -0.85 | -0.87 | 0.58 | 0.95* | 0.73 | -0.94* |
| HI4OMT | 0.51 | 0.28 | 0.45 | 0.34 | 0.47 | -0.71 | 0.95* | 0.86 | 0.99** | -0.16 | 1 | 0.08 | 0.37 | -0.24 | 0.34 | -0.32 | 0.19 | 0.06 |
| dxr | -0.63 | -0.77 | -0.68 | -0.76 | -0.68 | -0.25 | -0.07 | 0.57 | -0.05 | 0.93* | 0.08 | 1 | -0.6 | -0.81 | 0.45 | 0.77 | 0.92* | -0.8 |
| CHI | 0.98** | 0.97** | 0.98** | 0.97** | 0.29 | 0.32 | 0.63 | -0.08 | 0.51 | -0.85 | 0.37 | -0.6 | 1 | 0.79 | -0.71 | -0.97** | -0.25 | 0.93* |
| mvaK1 | 0.71 | 0.86 | 0.76 | 0.83 | 0.19 | 0.7 | 0.04 | -0.67 | -0.08 | -0.87 | -0.24 | -0.81 | 0.79 | 1 | -0.89* | -0.85 | -0.56 | 0.96* |
| HMGCR | -0.56 | -0.69 | -0.59 | -0.65 | 0.27 | -0.89* | 0.04 | 0.6 | 0.2 | 0.58 | 0.34 | 0.45 | -0.71 | -0.89* | 1 | 0.66 | 0.14 | -0.82 |
| CAS1 | -0.97** | -1.00** | -0.99** | -1.00** | -0.47 | -0.27 | -0.55 | 0.19 | -0.47 | 0.95* | -0.32 | 0.77 | -0.97** | -0.85 | 0.66 | 1 | 0.48 | -0.96** |
| 4CL | -0.32 | -0.47 | -0.37 | -0.47 | -0.76 | -0.06 | 0.15 | 0.57 | 0.1 | 0.73 | 0.19 | 0.92* | -0.25 | -0.56 | 0.14 | 0.48 | 1 | -0.51 |
| SQLE(SE) | 0.89* | 0.97** | 0.92* | 0.96* | 0.32 | 0.51 | 0.33 | -0.42 | 0.22 | -0.94* | 0.06 | -0.8 | 0.93* | 0.96* | -0.82 | -0.96** | -0.51 | 1 |

Note: Abbreviations Capital letters represent respectively: RL-Root length, RD-Root diameter, RFW-Root fresh weight, TLR-Thick lateral root, CIV-Content of astragaloside IV, CCG-Content of calycosin 7-O-β-D-glucopyranoside. **P*＜0.05, ***P*＜0.01.
