# Supplementary material for: Integrated phenotypic, transcriptomics and metabolomics: growth status and metabolite accumulation pattern of medicinal materials at different harvest periods of Astragalus Membranaceus Mongholicus
Source: BMC Plant Biol. 2024 May 3;24:358. doi: 10.1186/s12870-024-05030-7 (PMC11067282; doi:10.1186/s12870-024-05030-7)
Supplement: Supplementary file 16 — Additional file 16: Table S14. Pearson correlation analysis of 9 metabolites with the root phenotype and the content of the two main active components. [file 12870_2024_5030_MOESM16_ESM.docx]

Table S14. Pearson correlation analysis of 9 metabolites with the root phenotype and the content of the two main active components

|  | RL | RD | RFW | TLR | CIV | CCG | Isoliquiritigenin | Astragaloside III | Calycosin | Daidzein | Liquiritigenin | Formononetin | Mevalonate-5PP | L-Phenylalanine | Trans-Cinnamic Acid |
| --- | --- | --- | --- | --- | --- | --- | --- | --- | --- | --- | --- | --- | --- | --- | --- |
| RL | 1 | 0.97** | 1.00** | 0.98** | 0.46 | 0.13 | -0.97** | 0.25 | -0.87 | -0.53 | 0.68 | -0.22 | -0.93* | 0.02 | 0.64 |
| RD | 0.97** | 1 | 0.98** | 1.00** | 0.44 | 0.32 | -0.90* | 0.34 | -0.72 | -0.34 | 0.84 | -0.04 | -0.96** | -0.2 | 0.52 |
| RFW | 1.00** | 0.98** | 1 | 0.99** | 0.47 | 0.17 | -0.96* | 0.29 | -0.83 | -0.49 | 0.73 | -0.18 | -0.95* | -0.03 | 0.6 |
| TLR | 0.98** | 1.00** | 0.99** | 1 | 0.48 | 0.26 | -0.93* | 0.36 | -0.75 | -0.4 | 0.81 | -0.1 | -0.97** | -0.14 | 0.52 |
| CIV | 0.46 | 0.44 | 0.47 | 0.48 | 1 | -0.53 | -0.65 | 0.90* | -0.41 | -0.71 | 0.19 | -0.76 | -0.67 | 0.47 | -0.29 |
| CCG | 0.13 | 0.32 | 0.17 | 0.26 | -0.53 | 1 | 0.12 | -0.25 | 0.26 | 0.77 | 0.71 | 0.93* | -0.12 | -0.97** | 0.23 |
| Isoliquiritigenin | -0.97** | -0.90* | -0.96* | -0.93* | -0.65 | 0.12 | 1 | -0.4 | 0.90* | 0.71 | -0.54 | 0.46 | 0.93* | -0.25 | -0.51 |
| Astragaloside III | 0.25 | 0.34 | 0.29 | 0.36 | 0.90* | -0.25 | -0.4 | 1 | -0.03 | -0.32 | 0.34 | -0.45 | -0.58 | 0.11 | -0.57 |
| Calycosin | -0.87 | -0.72 | -0.83 | -0.75 | -0.41 | 0.26 | 0.90* | -0.03 | 1 | 0.8 | -0.24 | 0.51 | 0.68 | -0.45 | -0.75 |
| Daidzein | -0.53 | -0.34 | -0.49 | -0.4 | -0.71 | 0.77 | 0.71 | -0.32 | 0.8 | 1 | 0.19 | 0.93* | 0.46 | -0.85 | -0.27 |
| Liquiritigenin | 0.68 | 0.84 | 0.73 | 0.81 | 0.19 | 0.71 | -0.54 | 0.34 | -0.24 | 0.19 | 1 | 0.41 | -0.78 | -0.67 | 0.24 |
| Formononetin | -0.22 | -0.04 | -0.18 | -0.1 | -0.76 | 0.93* | 0.46 | -0.45 | 0.51 | 0.93* | 0.41 | 1 | 0.24 | -0.93* | 0.09 |
| Mevalonate-5PP | -0.93* | -0.96** | -0.95* | -0.97** | -0.67 | -0.12 | 0.93* | -0.58 | 0.68 | 0.46 | -0.78 | 0.24 | 1 | 0.06 | -0.31 |
| L-Phenylalanine | 0.02 | -0.2 | -0.03 | -0.14 | 0.47 | -0.97** | -0.25 | 0.11 | -0.45 | -0.85 | -0.67 | -0.93* | 0.06 | 1 | 0.02 |
| Trans-Cinnamic Acid | 0.64 | 0.52 | 0.6 | 0.52 | -0.29 | 0.23 | -0.51 | -0.57 | -0.75 | -0.27 | 0.24 | 0.09 | -0.31 | 0.02 | 1 |

Note: Abbreviations Capital letters represent respectively: RL-Root length, RD-Root diameter, RFW-Root fresh weight, TLR-Thick lateral root, CIV-Content of astragaloside IV, CCG-Content of calycosin 7-O-β-D-glucopyranoside. **P*＜0.05, ***P*＜0.01.

**Table S17 qRT–PCR primers used in this study**

| No. | KO name | Gene ID | Sequence (5'to3') |
| --- | --- | --- | --- |
| 1 | ACAT | TRINITY_DN3633_c0_g1 | F: CGTGGGAAATTGTTCCAGTT |
|  |  |  | R: TCCAAGCTTCAATGCCTTCT |
| 2 | HMGCR | TRINITY_DN7058_c1_g3 | F: TTCCCAATCAGTGCCGT |
|  |  |  | R: GGTAGATGAAGGAGGCGATG |
| 3 | mvaK1 | TRINITY_DN5828_c0_g1 | F: ATGAGGCACCAGGATGCTAT |
|  |  |  | R: GCTCCTGTCAATTTGGAAGC |
| 4 | IDI | TRINITY_DN1814_c0_g3 | F: GCTCGGTATTCCTGCTGAAG |
|  |  |  | R: CAGCTTCAAACCTCCCTCAC |
| 5 | dxr | TRINITY_DN4306_c0_g3 | F: TGGTCACGGGAATAGTAGGC |
|  |  |  | R: ATGGCAGAATGTTCGGAATC |
| 6 | SQLE(SE) | TRINITY_DN9930_c0_g2 | F: GGCAGGAGCACTTTACAAGG |
|  |  |  | R: CAATCCACATCCGCTTAGGT |
| 7 | CAS1 | TRINITY_DN8096_c0_g1 | F: TGAGGGACCTAATGACGGAC |
|  |  |  | R: GCATGTATGGAAGGAGCCAT |
| 8 | PAL | TRINITY_DN2877_c0_g1 | F: CATGCAAGGGAAACCTGAAT |
|  |  |  | R: ATCAAAGGACCAAGCCATTG |
| 9 | 4CL | TRINITY_DN855_c0_g1 | F: TTGCTGCAAAGGTTGTGG |
|  |  |  | R: ATTGGAACCAACATCGCC |
| 10 | CHS | TRINITY_DN12019_c0_g1 | F: TTTCGTGGCCCAAGTGA |
|  |  |  | R: TGGAGCGATTGTTTGTGC |
| 11 | CHI | TRINITY_DN5350_c0_g1 | F: CCAGGTTCCACCAAGTCCTA |
|  |  |  | R: GCTGGAGTCTTACCCTTCCA |
| 12 | HI4OMT | TRINITY_DN14288_c0_g1 | F: TTGACATGTGGCACTCTTCC |
|  |  |  | R: ATTCGAGAATCAGCAGCCAT |
|  | 18S RNA | - | F: TCAACCATAAACGATGCCGACC |
|  |  |  | R: TTTCAGCCTTGCGACCATACTCC |


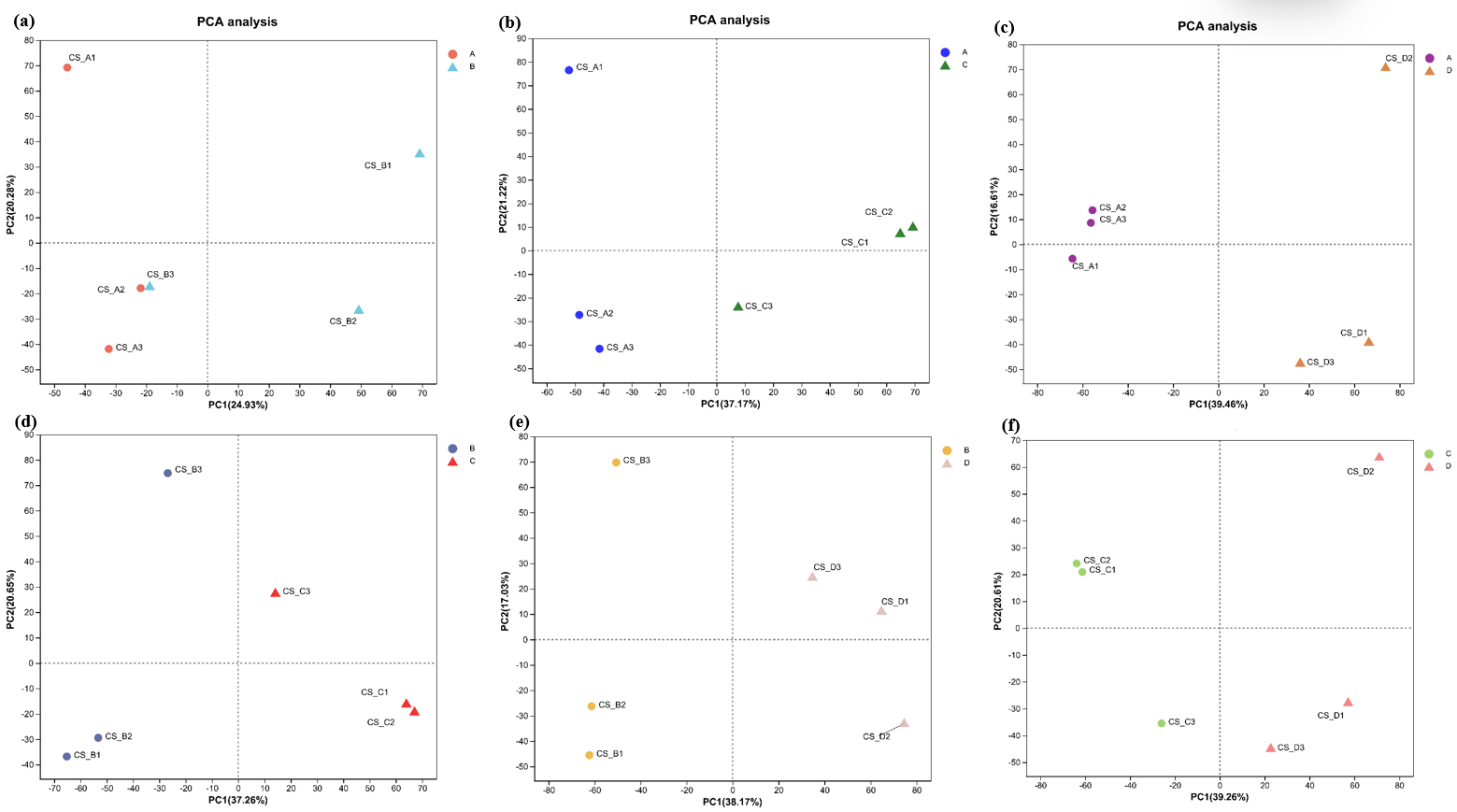


**Figure S1.** PCA plot of roots transcriptome profiles. (a) Stage A vs. B. (b) Stage A vs. C. (c) Stage A vs. D. (d) Stage B vs. C. (e) Stage B vs. D. (f) Stage C vs. D.


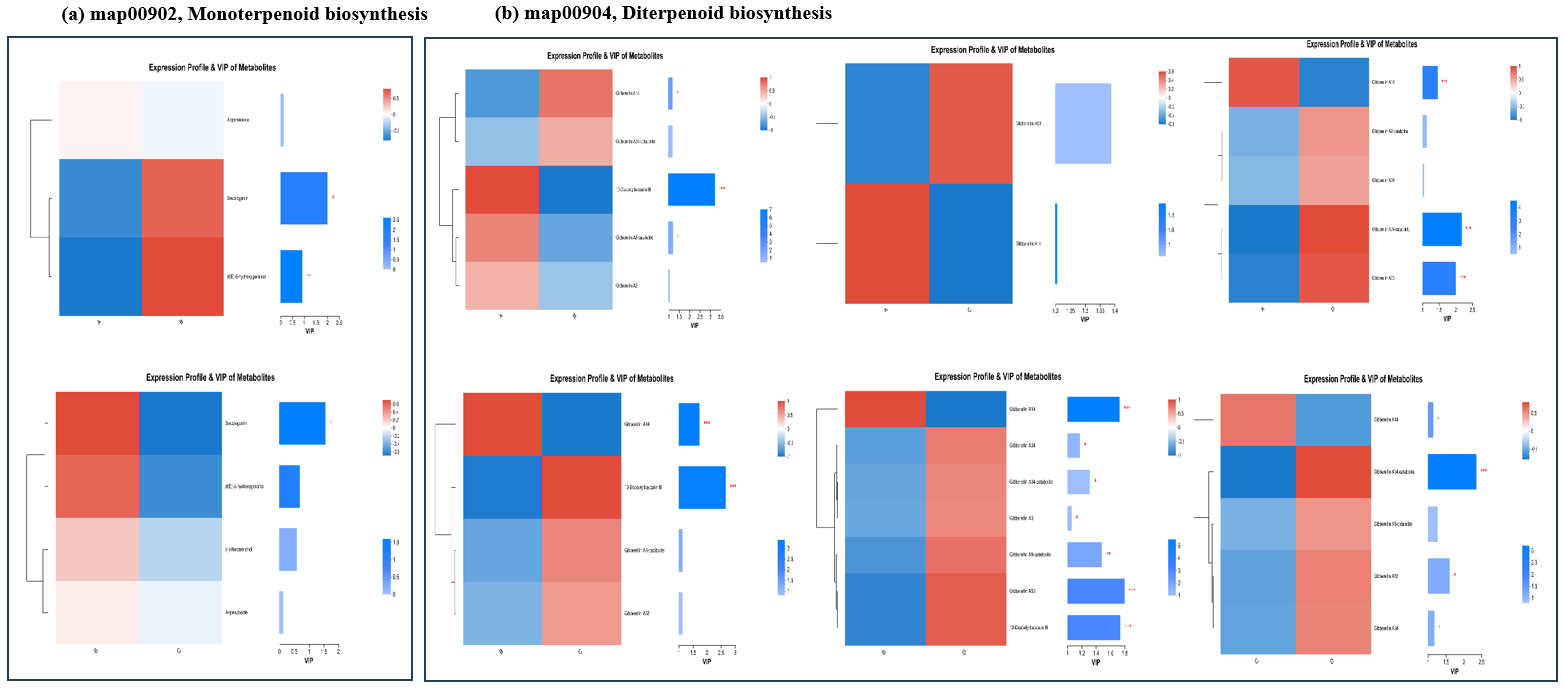


**Figure S2.** VIP scores analysis of monoterpenoids and diterpenoids. Statistical significance was determined by Student's t-test. * p < 0.05, ** p < 0.01.
