# Supplementary material for: Integrated phenotypic, transcriptomics and metabolomics: growth status and metabolite accumulation pattern of medicinal materials at different harvest periods of Astragalus Membranaceus Mongholicus
Source: BMC Plant Biol. 2024 May 3;24:358. doi: 10.1186/s12870-024-05030-7 (PMC11067282; doi:10.1186/s12870-024-05030-7)
Supplement: Supplementary file 17 — Additional file 17: Table S15. Pearson correlation analysis of 16 Degs and 9 metabolites by FPKM and UHPLC-Exactive HF-X. [file 12870_2024_5030_MOESM17_ESM.docx]

Table S15. Pearson correlation analysis of 16 Degs and 9 metabolites by FPKM and UHPLC-Exactive HF-X

|  | Isoliquiritigenin | Astragaloside | Calycosin | Daidzein | Liquiritigenin | Formononetin | Mevalonate-5PP | L-Phenylalanine | Trans-Cinnamic Acid | COMT | CSE | SUS | REF1 | CHS2 | CHS1 | CYP75A | CYP81E | PTR | CYP75B1 | HMGCS | HMGCR1 | mvaK1 | dxs | HMGCR2 | HMGCR3 |
| --- | --- | --- | --- | --- | --- | --- | --- | --- | --- | --- | --- | --- | --- | --- | --- | --- | --- | --- | --- | --- | --- | --- | --- | --- | --- |
| Isoliquiritigenin | 1 | -0.4 | 0.90* | 0.71 | -0.54 | 0.46 | 0.93* | -0.25 | -0.51 | 0.87 | -0.78 | -0.77 | 0.90* | -0.85 | -0.97** | -0.88 | -0.75 | -0.37 | -0.48 | 0.7 | 0.33 | -0.57 | 0.57 | 0.22 | 0.22 |
| Astragaloside III | -0.4 | 1 | -0.03 | -0.32 | 0.34 | -0.45 | -0.58 | 0.11 | -0.57 | -0.03 | 0.32 | 0.29 | -0.16 | 0.02 | 0.24 | 0.14 | 0.29 | -0.06 | 0.24 | 0.14 | 0.15 | 0.32 | 0.48 | 0.26 | 0.49 |
| Calycosin | 0.90* | -0.03 | 1 | 0.8 | -0.24 | 0.51 | 0.68 | -0.45 | -0.75 | 0.82 | -0.55 | -0.55 | 0.79 | -0.99** | -0.89* | -0.98** | -0.52 | -0.19 | -0.2 | 0.67 | 0.2 | -0.28 | 0.73 | 0.12 | 0.24 |
| Daidzein | 0.71 | -0.32 | 0.8 | 1 | 0.19 | 0.93* | 0.46 | -0.85 | -0.27 | 0.35 | -0.12 | -0.11 | 0.37 | -0.87 | -0.56 | -0.91* | -0.07 | 0.38 | 0.27 | 0.11 | -0.4 | 0.16 | 0.19 | -0.48 | -0.39 |
| Liquiritigenin | -0.54 | 0.34 | -0.24 | 0.19 | 1 | 0.41 | -0.78 | -0.67 | 0.24 | -0.72 | 0.94* | 0.94* | -0.78 | 0.08 | 0.65 | 0.1 | 0.95* | 0.92* | 0.99** | -0.77 | -0.88* | 1.00** | -0.43 | -0.82 | -0.66 |
| Formononetin | 0.46 | -0.45 | 0.51 | 0.93* | 0.41 | 1 | 0.24 | -0.93* | 0.09 | 0 | 0.17 | 0.18 | 0.03 | -0.62 | -0.25 | -0.69 | 0.22 | 0.66 | 0.5 | -0.27 | -0.68 | 0.4 | -0.18 | -0.76 | -0.71 |
| Mevalonate-5PP | 0.93* | -0.58 | 0.68 | 0.46 | -0.78 | 0.24 | 1 | 0.06 | -0.31 | 0.83 | -0.91* | -0.90* | 0.90* | -0.58 | -0.91* | -0.63 | -0.89* | -0.56 | -0.72 | 0.71 | 0.5 | -0.79 | 0.43 | 0.39 | 0.28 |
| L-Phenylalanine | -0.25 | 0.11 | -0.45 | -0.85 | -0.67 | -0.93* | 0.06 | 1 | 0.02 | 0.14 | -0.42 | -0.42 | 0.15 | 0.59 | 0.06 | 0.62 | -0.46 | -0.79 | -0.73 | 0.36 | 0.79 | -0.65 | 0.13 | 0.82 | 0.68 |
| Trans-Cinnamic Acid | -0.51 | -0.57 | -0.75 | -0.27 | 0.24 | 0.09 | -0.31 | 0.02 | 1 | -0.77 | 0.46 | 0.48 | -0.68 | 0.7 | 0.66 | 0.61 | 0.46 | 0.47 | 0.29 | -0.81 | -0.52 | 0.29 | -0.98** | -0.52 | -0.72 |
| COMT | 0.87 | -0.03 | 0.82 | 0.35 | -0.72 | 0 | 0.83 | 0.14 | -0.77 | 1 | -0.90* | -0.91* | 0.99** | -0.71 | -0.96** | -0.69 | -0.89* | -0.72 | -0.71 | 0.96** | 0.72 | -0.75 | 0.86 | 0.65 | 0.68 |
| CSE | -0.78 | 0.32 | -0.55 | -0.12 | 0.94* | 0.17 | -0.91* | -0.42 | 0.46 | -0.90* | 1 | 1.00** | -0.94* | 0.4 | 0.87 | 0.42 | 1.00** | 0.85 | 0.92* | -0.89* | -0.82 | 0.96* | -0.61 | -0.73 | -0.64 |
| SUS | -0.77 | 0.29 | -0.55 | -0.11 | 0.94* | 0.18 | -0.90* | -0.42 | 0.48 | -0.91* | 1.00** | 1 | -0.94* | 0.41 | 0.87 | 0.42 | 1.00** | 0.86 | 0.93* | -0.90* | -0.83 | 0.95* | -0.63 | -0.75 | -0.66 |
| REF1 | 0.90* | -0.16 | 0.79 | 0.37 | -0.78 | 0.03 | 0.90* | 0.15 | -0.68 | 0.99** | -0.94* | -0.94* | 1 | -0.68 | -0.97** | -0.68 | -0.93* | -0.72 | -0.76 | 0.94* | 0.71 | -0.8 | 0.78 | 0.62 | 0.62 |
| CHS2 | -0.85 | 0.02 | -0.99** | -0.87 | 0.08 | -0.62 | -0.58 | 0.59 | 0.7 | -0.71 | 0.4 | 0.41 | -0.68 | 1 | 0.81 | 0.99** | 0.37 | 0.03 | 0.04 | -0.54 | -0.03 | 0.12 | -0.65 | 0.04 | -0.11 |
| CHS1 | -0.97** | 0.24 | -0.89* | -0.56 | 0.65 | -0.25 | -0.91* | 0.06 | 0.66 | -0.96** | 0.87 | 0.87 | -0.97** | 0.81 | 1 | 0.82 | 0.84 | 0.55 | 0.61 | -0.85 | -0.53 | 0.68 | -0.73 | -0.44 | -0.45 |
| CYP75A | -0.88 | 0.14 | -0.98** | -0.91* | 0.1 | -0.69 | -0.63 | 0.62 | 0.61 | -0.69 | 0.42 | 0.42 | -0.68 | 0.99** | 0.82 | 1 | 0.38 | 0 | 0.04 | -0.5 | 0.01 | 0.13 | -0.57 | 0.09 | -0.03 |
| CYP81E | -0.75 | 0.29 | -0.52 | -0.07 | 0.95* | 0.22 | -0.89* | -0.46 | 0.46 | -0.89* | 1.00** | 1.00** | -0.93* | 0.37 | 0.84 | 0.38 | 1 | 0.88 | 0.94* | -0.89* | -0.85 | 0.97** | -0.62 | -0.77 | -0.67 |
| PTR | -0.37 | -0.06 | -0.19 | 0.38 | 0.92* | 0.66 | -0.56 | -0.79 | 0.47 | -0.72 | 0.85 | 0.86 | -0.72 | 0.03 | 0.55 | 0 | 0.88 | 1 | 0.95* | -0.85 | -1.00** | 0.92* | -0.63 | -0.98** | -0.90* |
| CYP75B1 | -0.48 | 0.24 | -0.2 | 0.27 | 0.99** | 0.5 | -0.72 | -0.73 | 0.29 | -0.71 | 0.92* | 0.93* | -0.76 | 0.04 | 0.61 | 0.04 | 0.94* | 0.95* | 1 | -0.79 | -0.92* | 0.99** | -0.47 | -0.87 | -0.73 |
| HMGCS | 0.7 | 0.14 | 0.67 | 0.11 | -0.77 | -0.27 | 0.71 | 0.36 | -0.81 | 0.96** | -0.89* | -0.90* | 0.94* | -0.54 | -0.85 | -0.5 | -0.89* | -0.85 | -0.79 | 1 | 0.86 | -0.8 | 0.90* | 0.82 | 0.85 |
| HMGCR1 | 0.33 | 0.15 | 0.2 | -0.4 | -0.88* | -0.68 | 0.5 | 0.79 | -0.52 | 0.72 | -0.82 | -0.83 | 0.71 | -0.03 | -0.53 | 0.01 | -0.85 | -1.00** | -0.92* | 0.86 | 1 | -0.89* | 0.67 | 0.99** | 0.93* |
| mvaK1 | -0.57 | 0.32 | -0.28 | 0.16 | 1.00** | 0.4 | -0.79 | -0.65 | 0.29 | -0.75 | 0.96* | 0.95* | -0.8 | 0.12 | 0.68 | 0.13 | 0.97** | 0.92* | 0.99** | -0.8 | -0.89* | 1 | -0.47 | -0.82 | -0.67 |
| dxs | 0.57 | 0.48 | 0.73 | 0.19 | -0.43 | -0.18 | 0.43 | 0.13 | -0.98** | 0.86 | -0.61 | -0.63 | 0.78 | -0.65 | -0.73 | -0.57 | -0.62 | -0.63 | -0.47 | 0.90* | 0.67 | -0.47 | 1 | 0.66 | 0.81 |
| HMGCR2 | 0.22 | 0.26 | 0.12 | -0.48 | -0.82 | -0.76 | 0.39 | 0.82 | -0.52 | 0.65 | -0.73 | -0.75 | 0.62 | 0.04 | -0.44 | 0.09 | -0.77 | -0.98** | -0.87 | 0.82 | 0.99** | -0.82 | 0.66 | 1 | 0.96* |
| HMGCR3 | 0.22 | 0.49 | 0.24 | -0.39 | -0.66 | -0.71 | 0.28 | 0.68 | -0.72 | 0.68 | -0.64 | -0.66 | 0.62 | -0.11 | -0.45 | -0.03 | -0.67 | -0.90* | -0.73 | 0.85 | 0.93* | -0.67 | 0.81 | 0.96* | 1 |

Note: **P*＜0.05, ***P*＜0.01.
