# Supplementary material for: Integrated phenotypic, transcriptomics and metabolomics: growth status and metabolite accumulation pattern of medicinal materials at different harvest periods of Astragalus Membranaceus Mongholicus
Source: BMC Plant Biol. 2024 May 3;24:358. doi: 10.1186/s12870-024-05030-7 (PMC11067282; doi:10.1186/s12870-024-05030-7)
Supplement: Supplementary file 18 — Additional file 18: Table S16. Pearson correlation analysis of 12 genes and 9 metabolites by FPKM and UHPLC-Exactive HF-X [file 12870_2024_5030_MOESM18_ESM.docx]

Table S16. Pearson correlation analysis of 12 genes and 9 metabolites by FPKM and UHPLC-Exactive HF-X

|  | Isoliquiritigenin | Astragaloside | Calycosin | Daidzein | Liquiritigenin | Formononetin | Mevalonate-5PP | L-Phenylalanine | Trans-Cinnamic Acid | CHS | IDI | PAL | ACAT | HI4OMT | dxr | CHI | mvaK1 | HMGCR | CAS1 | 4CL | SQLE(SE) |
| --- | --- | --- | --- | --- | --- | --- | --- | --- | --- | --- | --- | --- | --- | --- | --- | --- | --- | --- | --- | --- | --- |
| Isoliquiritigenin | 1 | -0.4 | 0.90* | 0.71 | -0.54 | 0.46 | 0.93* | -0.25 | -0.51 | -0.81 | -0.18 | -0.77 | 0.85 | -0.65 | 0.63 | -0.89* | -0.57 | 0.33 | 0.92* | 0.39 | -0.78 |
| Astragaloside III | -0.4 | 1 | -0.03 | -0.32 | 0.34 | -0.45 | -0.58 | 0.11 | -0.57 | -0.01 | -0.31 | 0.09 | -0.62 | 0.03 | -0.81 | 0.13 | 0.32 | 0.15 | -0.37 | -0.95* | 0.32 |
| Calycosin | 0.90* | -0.03 | 1 | 0.8 | -0.24 | 0.51 | 0.68 | -0.45 | -0.75 | -0.97** | -0.53 | -0.92* | 0.55 | -0.85 | 0.24 | -0.8 | -0.28 | 0.2 | 0.73 | -0.04 | -0.55 |
| Daidzein | 0.71 | -0.32 | 0.8 | 1 | 0.19 | 0.93* | 0.46 | -0.85 | -0.27 | -0.89* | -0.74 | -0.96* | 0.31 | -0.96* | 0.14 | -0.36 | 0.16 | -0.4 | 0.38 | 0.09 | -0.12 |
| Liquiritigenin | -0.54 | 0.34 | -0.24 | 0.19 | 1 | 0.41 | -0.78 | -0.67 | 0.24 | 0 | -0.7 | -0.11 | -0.86 | -0.27 | -0.82 | 0.77 | 1.00** | -0.88* | -0.83 | -0.59 | 0.94* |
| Formononetin | 0.46 | -0.45 | 0.51 | 0.93* | 0.41 | 1 | 0.24 | -0.93* | 0.09 | -0.66 | -0.71 | -0.78 | 0.11 | -0.83 | 0.06 | -0.02 | 0.4 | -0.68 | 0.09 | 0.16 | 0.17 |
| Mevalonate-5PP | 0.93* | -0.58 | 0.68 | 0.46 | -0.78 | 0.24 | 1 | 0.06 | -0.31 | -0.52 | 0.19 | -0.48 | 0.98** | -0.33 | 0.87 | -0.88* | -0.79 | 0.5 | 0.97** | 0.65 | -0.91* |
| L-Phenylalanine | -0.25 | 0.11 | -0.45 | -0.85 | -0.67 | -0.93* | 0.06 | 1 | 0.02 | 0.65 | 0.90* | 0.76 | 0.21 | 0.85 | 0.3 | -0.15 | -0.65 | 0.79 | 0.16 | 0.2 | -0.42 |
| Trans-Cinnamic Acid | -0.51 | -0.57 | -0.75 | -0.27 | 0.24 | 0.09 | -0.31 | 0.02 | 1 | 0.67 | 0.36 | 0.54 | -0.22 | 0.48 | 0.14 | 0.7 | 0.29 | -0.52 | -0.51 | 0.51 | 0.45 |
| COMT | -0.81 | -0.01 | -0.97** | -0.89* | 0 | -0.66 | -0.52 | 0.65 | 0.67 | 1 | 0.72 | 0.98** | -0.37 | 0.95* | -0.07 | 0.63 | 0.04 | 0.04 | -0.55 | 0.15 | 0.33 |
| CSE | -0.18 | -0.31 | -0.53 | -0.74 | -0.7 | -0.71 | 0.19 | 0.90* | 0.36 | 0.72 | 1 | 0.77 | 0.36 | 0.86 | 0.57 | -0.08 | -0.67 | 0.6 | 0.19 | 0.57 | -0.42 |
| SUS | -0.77 | 0.09 | -0.92* | -0.96* | -0.11 | -0.78 | -0.48 | 0.76 | 0.54 | 0.98** | 0.77 | 1 | -0.31 | 0.99** | -0.05 | 0.51 | -0.08 | 0.2 | -0.47 | 0.1 | 0.22 |
| REF1 | 0.85 | -0.62 | 0.55 | 0.31 | -0.86 | 0.11 | 0.98** | 0.21 | -0.22 | -0.37 | 0.36 | -0.31 | 1 | -0.16 | 0.93* | -0.85 | -0.87 | 0.58 | 0.95* | 0.73 | -0.94* |
| CHS2 | -0.65 | 0.03 | -0.85 | -0.96* | -0.27 | -0.83 | -0.33 | 0.85 | 0.48 | 0.95* | 0.86 | 0.99** | -0.16 | 1 | 0.08 | 0.37 | -0.24 | 0.34 | -0.32 | 0.19 | 0.06 |
| CHS1 | 0.63 | -0.81 | 0.24 | 0.14 | -0.82 | 0.06 | 0.87 | 0.3 | 0.14 | -0.07 | 0.57 | -0.05 | 0.93* | 0.08 | 1 | -0.6 | -0.81 | 0.45 | 0.77 | 0.92* | -0.8 |
| CYP75A | -0.89* | 0.13 | -0.8 | -0.36 | 0.77 | -0.02 | -0.88* | -0.15 | 0.7 | 0.63 | -0.08 | 0.51 | -0.85 | 0.37 | -0.6 | 1 | 0.79 | -0.71 | -0.97** | -0.25 | 0.93* |
| CYP81E | -0.57 | 0.32 | -0.28 | 0.16 | 1.00** | 0.4 | -0.79 | -0.65 | 0.29 | 0.04 | -0.67 | -0.08 | -0.87 | -0.24 | -0.81 | 0.79 | 1 | -0.89* | -0.85 | -0.56 | 0.96* |
| PTR | 0.33 | 0.15 | 0.2 | -0.4 | -0.88* | -0.68 | 0.5 | 0.79 | -0.52 | 0.04 | 0.6 | 0.2 | 0.58 | 0.34 | 0.45 | -0.71 | -0.89* | 1 | 0.66 | 0.14 | -0.82 |
| CYP75B1 | 0.92* | -0.37 | 0.73 | 0.38 | -0.83 | 0.09 | 0.97** | 0.16 | -0.51 | -0.55 | 0.19 | -0.47 | 0.95* | -0.32 | 0.77 | -0.97** | -0.85 | 0.66 | 1 | 0.48 | -0.96** |
| HMGCS | 0.39 | -0.95* | -0.04 | 0.09 | -0.59 | 0.16 | 0.65 | 0.2 | 0.51 | 0.15 | 0.57 | 0.1 | 0.73 | 0.19 | 0.92* | -0.25 | -0.56 | 0.14 | 0.48 | 1 | -0.51 |
| HMGCR1 | -0.78 | 0.32 | -0.55 | -0.12 | 0.94* | 0.17 | -0.91* | -0.42 | 0.45 | 0.33 | -0.42 | 0.22 | -0.94* | 0.06 | -0.8 | 0.93* | 0.96* | -0.82 | -0.96** | -0.51 | 1 |

Note: **P*＜0.05, ***P*＜0.01
