# Supplementary material for: Integrated phenotypic, transcriptomics and metabolomics: growth status and metabolite accumulation pattern of medicinal materials at different harvest periods of Astragalus Membranaceus Mongholicus
Source: BMC Plant Biol. 2024 May 3;24:358. doi: 10.1186/s12870-024-05030-7 (PMC11067282; doi:10.1186/s12870-024-05030-7)
Supplement: Supplementary file 19 — Additional file 19: Table S17. qRT–PCR primers used in this study. [file 12870_2024_5030_MOESM19_ESM.docx]

**Table S17 qRT–PCR primers used in this study**

| No. | KO name | Gene ID | Sequence (5'to3') |
| --- | --- | --- | --- |
| 1 | ACAT | TRINITY_DN3633_c0_g1 | F: CGTGGGAAATTGTTCCAGTT |
|  |  |  | R: TCCAAGCTTCAATGCCTTCT |
| 2 | HMGCR | TRINITY_DN7058_c1_g3 | F: TTCCCAATCAGTGCCGT |
|  |  |  | R: GGTAGATGAAGGAGGCGATG |
| 3 | mvaK1 | TRINITY_DN5828_c0_g1 | F: ATGAGGCACCAGGATGCTAT |
|  |  |  | R: GCTCCTGTCAATTTGGAAGC |
| 4 | IDI | TRINITY_DN1814_c0_g3 | F: GCTCGGTATTCCTGCTGAAG |
|  |  |  | R: CAGCTTCAAACCTCCCTCAC |
| 5 | dxr | TRINITY_DN4306_c0_g3 | F: TGGTCACGGGAATAGTAGGC |
|  |  |  | R: ATGGCAGAATGTTCGGAATC |
| 6 | SQLE(SE) | TRINITY_DN9930_c0_g2 | F: GGCAGGAGCACTTTACAAGG |
|  |  |  | R: CAATCCACATCCGCTTAGGT |
| 7 | CAS1 | TRINITY_DN8096_c0_g1 | F: TGAGGGACCTAATGACGGAC |
|  |  |  | R: GCATGTATGGAAGGAGCCAT |
| 8 | PAL | TRINITY_DN2877_c0_g1 | F: CATGCAAGGGAAACCTGAAT |
|  |  |  | R: ATCAAAGGACCAAGCCATTG |
| 9 | 4CL | TRINITY_DN855_c0_g1 | F: TTGCTGCAAAGGTTGTGG |
|  |  |  | R: ATTGGAACCAACATCGCC |
| 10 | CHS | TRINITY_DN12019_c0_g1 | F: TTTCGTGGCCCAAGTGA |
|  |  |  | R: TGGAGCGATTGTTTGTGC |
| 11 | CHI | TRINITY_DN5350_c0_g1 | F: CCAGGTTCCACCAAGTCCTA |
|  |  |  | R: GCTGGAGTCTTACCCTTCCA |
| 12 | HI4OMT | TRINITY_DN14288_c0_g1 | F: TTGACATGTGGCACTCTTCC |
|  |  |  | R: ATTCGAGAATCAGCAGCCAT |
|  | 18S RNA | - | F: TCAACCATAAACGATGCCGACC |
|  |  |  | R: TTTCAGCCTTGCGACCATACTCC |
